# Expanding Roles for CD4 T Cells and Their Subpopulations in Tumor Immunity and Therapy

**DOI:** 10.3389/fonc.2013.00063

**Published:** 2013-03-26

**Authors:** Mark J. Dobrzanski

**Affiliations:** ^1^Department of Internal Medicine, Texas Tech University Health Sciences Center School of MedicineAmarillo, TX, USA

**Keywords:** CD4 helper cells, memory T cells, T regulatory cells, immunotherapy, cytolytic CD4 T cells, cytokines, tumor immunity

## Abstract

The importance of CD4 T cells in orchestrating the immune system and their role in inducing effective T cell-mediated therapies for the treatment of patients with select established malignancies are undisputable. Through a complex and balanced array of direct and indirect mechanisms of cellular activation and regulation, this functionally diverse family of lymphocytes can potentially promote tumor eradication, long-term tumor immunity, and aid in establishing and/or rebalancing immune cell homeostasis through interaction with other immune cell populations within the highly dynamic tumor environment. However, recent studies have uncovered additional functions and roles for CD4 T cells, some of which are independent of other lymphocytes, that can not only influence and contribute to tumor immunity but paradoxically promote tumor growth and progression. Here, we review the recent advances in our understanding of the various CD4 T cell lineages and their signature cytokines in disease progression and/or regression. We discuss their direct and indirect mechanistic interplay among themselves and with other responding cells of the antitumor response, their potential roles and abilities for “plasticity” and memory cell generation within the hostile tumor environment, and their potentials in cancer treatment and immunotherapy.

## Introduction

Cancer cells express antigens that differentiate them from their non-transformed counterparts (Van Der Bruggen et al., 2002). These antigens are often products of mutated cellular genes, aberrantly expressed normal genes, or genes encoding viral proteins. Categories and examples of human tumor antigens thus far identified include (a) the differentiation antigens encoded by genes that are only expressed in particular types of tissue such as those antigens expressed in melanocytes and melanoma and involved in melanin production, (b) mutational antigens that arise as a consequence of gene rearrangement and point mutations (i.e., p53), (c) cellular antigens that are over expressed in transformed cells when compared with their normal counterparts (i.e., HER-2), (d) molecules that display abnormal post-translational modifications (i.e., MUC1), (e) viral antigens derived from viral oncogenes (i.e., human papillomavirus proteins), and (f) cancer/testis (CT) antigens that are expressed in germ cells of testis and ovary but silent in normal somatic cells (i.e., MAGE and NY-ESO-1) (Cheever et al., 2009). It has been shown that tumors co-expressing such antigens can be recognized by effector T cells of the adaptive immune system and induce antitumor immune responses in both experimental animal and human systems (Dougan and Dranoff, 2009).

One of the earliest processes involved in the development of the adaptive immune response and tumor immunity is inflammation which functions to localize and eradicate tissue stressors induced by tumor growth and re-establish normal tissue homeostasis (Medzhitov, 2008). However, evidence obtained from various murine tumor models and clinical studies in cancer patients, have shown that chronic inflammation, mediated by ensuing adaptive immune responses, can contribute to tumorigenesis at all stages. For example, such responses may contribute to cancer initiation by generating genotoxic stress, to cancer promotion by inducing cellular proliferation, and to cancer progression by enhancing angiogenesis and tissue invasion (Grivennikov et al., 2010). In either instance, this forms the conceptual framework of the cancer immunoediting hypothesis, which stresses the dual host-protective and tumor-promoting actions of immunity on developing tumors (Schreiber et al., 2011; Vesely et al., 2011). In its most simplistic form, this model proposes that tumors develop through three successive and distinct phases termed “elimination,” “equilibration,” and “escape.” The elimination phase entails the process where the innate and adoptive immune systems collaboratively detect the presence of a developing tumor and destroy it before it becomes clinically apparent. The next phase of equilibration entails the process where rare tumor cell variants survive immune-mediated elimination and enter a state of equilibrium with the adaptive immune response. In this state, the immune system maintains residual tumor cells in a functional state of dormancy, a term used to describe latent tumor cells that may reside in patients for decades before eventually resuming growth as either recurrent primary tumors or distant metastases (Aguirre-Ghiso, 2007). Aside from preventing tumor outgrowth, it is also believed that the immune response in this phase “fashion” the immunogenicity of the occult tumors. Lastly, in the escape phase, tumor cells that have acquired the ability to circumvent immune recognition and/or destruction emerge as progressively growing and detectable tumors. It is further postulated that the course and progression through such phases are influenced and determined, in part, by tumor cell population changes in response to the immune system, host immune system changes in response to mechanisms re-establishing cellular homeostasis, or increased cancer-mediated immunosuppression and/or immune system decline. Such local and systemic environmental stressors are thought to be the major contributors to affect not only tumor outgrowth but also immunotherapeutic interventions and their efficacy in cancer patients. Thus, the tumor-promoting inflammation and protective tumor immunity processes appear dynamically interconnected where an imbalance can further result in the shaping of tumor immunogenicity which may either initiate and/or facilitate disease progression or regression.

At the earliest stages of the antitumor immune response, professional antigen presenting cells (APCs), most notably dendritic cells (DCs), encounter and capture tumor antigens that are released from either viable or dying tumor cells. This results in the activation and up-regulation of co-stimulatory molecules that facilitate and/or promote the migration of these cells to secondary lymphoid organs such as regional draining lymph nodes (Steinman and Mellman, 2004; Steinman and Banchereau, 2007). To develop into potent CD4 effector T cells that contribute to the antitumor immune response, naïve CD4 T cells need to recognize peptide antigens presented in an immunogenic context with HLA class II molecules on activated DCs. Additional co-stimulatory signals, such as DC-derived cytokines, then promote their differentiation into effector CD4 Th cell subsets characterized by distinct cytokine secreting profiles (O’Shea and Paul, 2010; Zhu et al., 2010). The best characterized of these effector cell subsets are the Th1 and Th2 cells, which are characterized by their production of IFN-γ and interleukin-4 (IL-4), respectively. More recently, the pro-inflammatory Th9 and Th17 cell subsets have also been shown to develop and/or reside in some tumors, along with TReg cells which are responsible for immune regulation and tissue homeostasis. Following recognition of a specific tumor-associated antigen presented by an appropriately activated APC, naïve CD4 T cells undergo several rounds of division and can become polarized into such distinct effector Th cell subsets that can differentially orchestrate antitumor immune responses, in part, through their production of signature cytokines. The differentiation of polarized CD4 effector T cells is controlled by unique sets of transcription factors, the expression of which is determined by multiple signals, in particular, by soluble factors that act on responding CD4 T cells during their activation. Subsequently, such differentiation results in distinct Th cell subsets characterized, in part, by select cytokine production that can initiate, facilitate, and influence distinct mechanistic arms of the immune response to tumors as determined in several murine tumor models as well as in evidence obtained from human studies. Although the production, function, and mechanistic interplay of these signature cytokines derived from the distinct Th cell subsets will be discussed in more detail below, it is becoming increasingly clear that considerable plasticity exists among the various subsets *in vivo*, especially during responses to tumors at various stages of development, progression, and/or regression. Moreover, certain cytokines such as IL-10, can be produced by nearly all subpopulations of cells within the multiple effector cell subsets further suggesting, that CD4 T cell responses are apparently convoluted and capable of initiating and maintaining quantitatively and qualitatively variable antitumor responses involved in facilitating either direct or indirect tumor cell killing or survival.

The CD4+ T cell represents a major component of the adaptive immune response and has been shown to be an integral part in the activation and regulation processes of the host response to many pathogens. Although the role of CD4 T cells in the antitumor response remains under investigated, it is becoming clear that effective immune responses to a developing or progressing tumor requires their activation, maturation, and active participation (Pardoll and Topalian, 1998; Blattman and Greenberg, 2004; Kennedy and Celis, 2008; Muranski and Restifo, 2009). As one of their primary emerging roles as “regulators” of the immune response to cancer, CD4 T cells have been shown to orchestrate and coordinate many facets of both the innate and adaptive immune responses to ensure optimal responses by other lymphocytes. CD4 T cells are necessary elements for priming tumor-specific CD8 T cells, influencing the differentiation and expansion of tumor antigen-specific CTLs and are essential for generating and maintaining long-term CD8 memory T cell responses (Janssen et al., 2003; Shedlock and Shen, 2003; Sun and Bevan, 2003; Sun et al., 2004). Moreover, several studies have defined additional roles for CD4 T cells, some of which are independent of other lymphocytes, that influence and/or contribute to tumor immunity during carcinogenesis. Paradoxically, several experimental and clinical observations have recently shown that these same CD4 effector cell subsets and their signature cytokines can not only contribute to antitumor responses but also tumor-promoting activities. We discuss what is known about the T cell subsets that develop during different stages of tumor growth and progression, how such diverse T cell subset responses contribute to disease progression and/or regression, their development into memory cells, and their potentials in cancer treatment.

## CD4 Effector T Cell Subsets

The roles of polarized CD4 T cell subsets in the antitumor immune response are greatly influenced by their signature cytokines which arm the cells with distinct immunological functions. Such cellular polarization processes are dependent, in part, on their expression of specific transcription factors that are influenced by multiple cellular and soluble biological signals within the priming milieu of the tumor environment. Moreover, substantial proportions of effector T cell subsets that are found *in vivo* are often characterized by plasticity and heterogeneity in terms of their cytokine-producing potentials. Thus effective tumor immunity is often dependent on such complex CD4 T cell responses following polarization and their interactions with other Th cell subsets within the hostile tumor environment. In any instance, the most characterized CD4 Th cell subset is the *Th1* that can potentially produce large amounts of IFN-γ upon tumor antigen encounter and expresses the transcription factor T-bet. The Th1 developmental pathway is typically driven by IL-12 activation of the signal transducer and activator of transcription 4 (STAT 4) and T-bet transcription factors during immune activation of naïve T cells (Szabo et al., 2000, 2003). As the “critical regulator” of the Th1 differentiation program, T-bet is responsible for the up-regulation of the IL-12 receptor β2 (IL-12β2R) subunit and confers IL-12 responsiveness and sustained T-bet expression (Lazarevic and Glimcher, 2011). In addition, it induces and upregulates IFN-γ (ifnγ) production but also induces the expression of genes encoding the chemokine receptor CXCR3 and the chemokines CCL3 and CCL4 (Jenner et al., 2009) which are responsible for enhancing the mobilization of select type 1-related immune cell responses to sites of tumor growth. In addition, T-bet suppresses commitment to the Th2 and Th17 lineage programs (Hwang et al., 2005). Although IFN-γ is considered the signature cytokine for this subset in both murine and human effector T cells, other cytokines have been shown to be produced by human Th1 cells and include IL-2, TNF-α, and IL-10. Interestingly, the importance of IL-10 production by Th1 effector cell subpopulations in the antitumor response is controversial. Several recent studies have suggested that IL-10 plays a role in inhibiting tumor development, growth, and metastases (Mocellin et al., 2005; Emmerich et al., 2012; Tanikawa et al., 2012). Whereas others have suggested that Th1 effector cell responses are auto-regulated through a negative feedback loop via the co-induction and expression of IL-10 (Cope et al., 2011). Conceivably, the relative amounts and/or duration of IFN-γ and IL-10 produced by such double-positive cytokine secreting Th1 cell subsets and their ability for “cytokine switching” might define the inflammatory/immune response, tolerance induction, and/or prevention of excessive immunopathology within the tumor microenvironment.

Th2 effector cell subsets are characterized by the production of IL-4, IL-5, and IL-13 and are responsible for coordinating humoral immunity and allergic inflammatory responses. IL-4 is primarily accountable for the differentiation of Th2 cells through STAT 6 and the transcription factor GATA-3 (Kaplan et al., 1996; Zheng and Flavell, 1997; Kurata et al., 1999; Zhu et al., 2001). The Th1 and Th2 developmental pathways among naïve CD4 T cells are controlled by a delicate balance of positive feedback loops, as IFN-γ enhances further Th1 development and IL-4 supports continued Th2 differentiation. At the same time, cross regulation by IFN-γ and IL-4 suppresses Th2 and Th1 differentiation, respectively. In a murine lung metastases model, Th2 effector cells have shown some indirect antitumor activity through the eosinophil chemotactic factor, eotaxin and eosinophil tumor infiltration (Mattes et al., 2003). However, the role of Th2 effector cells in the antitumor immune response remains unclear with several studies suggesting that such CD4 effector cells are associated with carcinogenesis and tumor progression (Tatsumi et al., 2002; Ochi et al., 2012). Recent investigations have shown that in addition to IL-10, which is essentially produced by all Th cell subsets, a subpopulation within the Th2 subset can preferentially co-produce IL-24 (a unique member of the IL-10 cytokine family) (Schaefer et al., 2001; Ouyang et al., 2011). Although its detailed regulation in Th2 cells is currently unclear, IL-24 has been shown to lack immune repressive functions, suppress human ovarian cancer cell growth both *in vitro* and *in vivo*, and induce substantial “bystander antitumor” immunity in patients (Fisher, 2005; Lebedeva et al., 2007; Emdad et al., 2009; Dash et al., 2010). Further investigation into understanding the development and properties of IL-24-secreting Th2 cells may provide profound therapeutic benefits for cancer patients.

The expression of IL-17 characterizes a subset of CD4 helper T cells (Th17). This cell lineage represents a third effector arm of CD4-mediated immune response and complements, in part, the functions of the Th1 and Th2 cell lineages. In addition to IL-17A and IL-17F, human Th17 cells also produce other cytokines such as IL-21, IL-22, and IL-26. In addition, the chemokine receptor CCR6 [which binds to the chemokine CC ligand 20 (CCL20) that is present in many malignant pleural effusions of lung cancer patients] is highly expressed on Th17 cells thus further facilitating their recruitment to sites of tumor growth and inflammation (Acosta-Rodriguez et al., 2007; Annunziato et al., 2007, 2012). Both human and murine Th17 cells express the transcription factors retinoic acid orphan receptor (ROR)γt (*Rorc*). ROR-γt is a critical regulator of Th17 cell differentiation and induces IL-17A, IL-17F, IL-26, and CCR6 expression while downregulating IFN-γ production in human naïve T cells (Manel et al., 2008). For induction and differentiation of murine Th17 cells, TGF-β and IL-6 are the most crucial cytokines for naïve CD4 cell differentiation (Murugaiyan and Saha, 2009; Gaffen, 2011). However, in the development of human Th17 cells, it has been shown that IL-6 and IL-1β, but not TGF-β, is essential for differentiation (Acosta-Rodriguez et al., 2007; Wilson et al., 2007). In any instance, IL-21 produced by Th17 cells further amplify Th17 generation in an autocrine manner and induce IL-23 receptor expression that enables cell responses to IL-23 stimulation. Subsequently, DC-derived IL-23 stabilizes the Th17 phenotype and helps Th17 cells to acquire effector functions. The induction of ROR-γt is dependent on STAT-3, which is preferentially activated by IL-6, IL-21, and IL-23. STAT-3 affects ROR-γt expression and binds to the IL-17 and IL-21 promoters. Thus, both STAT-3 and ROR-γt transcription factors regulate IL-17 production in a highly coordinated manner (Murugaiyan and Saha, 2009). In addition to characterizing Th17 cells by transcription factors and cytokine production, recent studies have shown that human Th17 cells originate from a CD4 precursor cell population that is present in both thymus and umbilical cord blood and co-expresses a member of the NK cell receptor-P1 family, CD161 (Cosmi et al., 2008). Although the precise function of CD161 is unknown at this time, it is considered a “hallmark” of human memory Th17 cells at sites of tissue inflammation (Cosmi et al., 2008; Kleinschek et al., 2009). Another marker that was identified to be specifically associated with human Th17 cells is the IL-4-induced gene 1 (IL-4I1) which encodes the enzyme l-phenylalanine oxidase. This enzyme is responsible, in part, for H_2_O_2_ production that can contribute to the inhibition of T lymphocyte proliferation (Boulland et al., 2007). As will be discussed below, Th17 cells are found in several human tumors. Although Th17-associated cytokines have been linked with carcinogenesis and tumor progression within the context of chronic inflammation and infection, it is becoming clear that this cell lineage can also contribute to the antitumor response in human malignancies of epithelial origin (Kryczek et al., 2007, 2009a; Zou and Restifo, 2010; Wilke et al., 2011).

Th22 CD4 T cells, which are thought to be a distinct Th subset that produce IL-22 independently of IL-17, were initially identified in patients with inflammatory disorders of the skin, intestine, and joints (Eyerich et al., 2009; Sonnenberg et al., 2011). More recently, such cells have also been identified and suggested to contribute to immune responses and inflammation in malignant pleural effusions and other human cancers (Zhang et al., 2008; Jiang et al., 2011; Miyagaki et al., 2011; Ye et al., 2012). Such Th22 cells express the cutaneous lymphocyte antigen (CLA), a functional E-selectin ligand that is involved in lymphocyte rolling on the endothelial cells of cutaneous postcapillary venules, along with the chemokine receptors CCR6, CCR4, and CCR10 which together facilitate the constitutive migration potentials of these cells to sites of inflammation (Duhen et al., 2009). Although the role, if any, of Th22 cells in the antitumor immune response is unclear, it is believed that such IL-22 producing CD4 Th cells contribute to local immune homeostasis and inflammation (Duhen et al., 2009).

The latest addition to the list of subsets, termed Th9, secretes IL-9 as the signature cytokine and may play a role in several inflammatory disorders. Naïve human and murine CD4 T cells both acquire a Th9 phenotype *in vitro* with the combination of IL-4 and TGF-β (Houssiau et al., 1995; Veldhoen et al., 2008; Beriou et al., 2010; Chang et al., 2010; Putheti et al., 2010; Yao et al., 2011; Jabeen and Kaplan, 2012; Wilhelm et al., 2012). The transcription factors STAT6, interferon regulatory factor 4 (IRF4), and PU.1 contribute to Th9 differentiation (Jabeen and Kaplan, 2012). IL-4-activated STAT6 promotes expression of IRF4 whereas PU.1 activation occurs downstream of TGF-β signaling where it directly bind to the IL-9 (*il9*) promoter (Chang et al., 2005, 2009, 2010). Interestingly, polarized Th2 cells can be deviated into Th9 cells by exposure to TGF-β, which results in down-regulation of GATA-3 and loss of IL-4 and IL-5 production. Using various gene knockout and transgenic mouse systems, Th9 cells have been shown to contribute to autoimmune and allergic inflammation processes (Noelle and Nowak, 2009). However, one recent study identified Th9 cells in healthy human blood and skin and also in metastatic lesions of patients with stage IV melanoma (Purwar et al., 2012). Moreover, using a murine tumor model, these same investigators showed that adoptive transfer of tumor-reactive Th9 cells and administration of recombinant IL-9 can effectively reduce melanoma growth. This evidence from a single study suggests that IL-9 producing Th9 cells may play a role in the generation of effective antitumor responses.

The final CD4 Th cell lineage to be described here resides in proximity to B cells within germinal centers of lymphoid tissues. These cells, referred to as follicular helper T cells (T_FH_), are responsible for providing B cell help and supporting B cell expansion and differentiation (Reinhardt et al., 2009; Ma et al., 2012). They are defined by expression of the transcription factor Bcl6 and the cytokines IL-21 (Liu et al., 2012b). Moreover, they express the chemokine receptor CXCR5 which facilitates migration to the B cell follicles after activation (Chtanova et al., 2004; Haynes et al., 2007). Studies have also shown that they possess key surface receptor molecules (PD-1, CD40 ligand, OX-40, CD84, and ICOS) that play critical roles in promoting B cell activation, differentiation, and survival (Ma et al., 2012). However, the role of T_FH_ cells in tumor immunity remains relatively undefined.

Accumulating evidence suggests that select CD4 effector T cell subsets may have a more “direct” role in inhibiting tumor growth and progression that are independent of their more “indirect” helper activities. As such, CD4 effector T cells have, in general, been shown to protect against both tumors and virally infected target cells through two distinct primary effector mechanisms. They include the production of cytokines, most notably IFN-γ and TNF (Hung et al., 1998; Pardoll and Topalian, 1998) and through direct cytolytic activity (Trapani and Smyth, 2002) that is mediated by degranulation of cytotoxic granules containing toxic effector molecules (i.e., perforin and granzyme) or ligation of the Fas (also known as CD95)/Fas Ligand (FasL; also known as CD95L) apoptotic killing pathway (Green and Ferguson, 2001). *Cytolytic CD4* T cells possessing such cytotoxic activity have been described in peripheral blood of both healthy and virally infected individuals (Feighery and Stastny, 1979; Appay et al., 2002; van Leeuwen et al., 2004; Casazza et al., 2006; Stuller and Flano, 2009; Nemes et al., 2010; Stuller et al., 2010). Phenotypic analysis has shown that they are CD45RO^+^, CCR7^−^, CD27^−^, and CD28^−^ and shown to possess high levels of the cytolytic effector molecules granzyme A, granzyme B and perforin (Appay et al., 2002; Casazza et al., 2006) suggesting that these cells are antigen-experienced and terminally differentiated CD4 effector cells. Moreover, as with CD8 effector T cells (Pearce et al., 2003; Intlekofer et al., 2005), there is evidence that expression of the eomesodermin (*Eomes*) transcription factor may be crucial in driving the development of cytotoxic CD4 T cells *in vivo* (Qui et al., 2011; Hirschhorn-Cymerman et al., 2012). Although T cell expression of eomesodermin has been linked to terminal differentiation and memory cell phenotype with the concomitant secretion of Th1 cytokines (Hirschhorn-Cymerman et al., 2012), others have suggested that cytotoxic activity of CD4 effector T cells does not depend on Th1 cell polarization (Brown et al., 2009). Thus suggesting that such cells constitute a unique and separate cell lineage from those already described. In either instance, more recent studies using a murine transgenic tumor model of advanced melanoma, showed that transfer of naïve tumor-reactive CD4 T cells into lymphopenic recipients can induce a substantial expansion and differentiation of Th1 cells with cytotoxic activity (Quezada et al., 2010; Xie et al., 2010). Moreover, induction of such cells correlated with class II-restricted tumor rejection that was dependent on the presence of IFN-γ which was believed to mediate the up-regulation of class II on tumor target cells. In more recent studies using both human and murine cells, generation of tumor-reactive cytotoxic CD4 Th1 cells were further shown to be induced, in part, by both the engagement of a specific co-stimulatory pathway of the tumor necrosis factor receptor (TNFR) family member, OX-40 (also known as CD134) and an intracellular mechanism relying on eomesodermin expression (Qui et al., 2011; Hirschhorn-Cymerman et al., 2012). Further identification and characterization of the mechanisms involved in the induction of tumor-reactive CD4 T cells with cytotoxic activities in cancer patients may offer significant advantages for future treatment strategies of human malignancies.

Lastly, the subpopulations of CD4^+^ TReg cells can be classified into two main subsets according to their origin and suppressive activity. Natural CD4^+^ TReg effector cells (nTRegs), constitutively expressing FoxP3 and the activation marker CD25, originate in the thymus by high affinity interaction of the T cell receptor (TCR) with Ag expressed on the thymic stroma (Sakaguchi, 2008; Shevach, 2009; Buckner, 2010; Nishikawa and Sakaguchi, 2010; Sakaguchi et al., 2010; Miyara and Sakaguchi, 2011). Such cells suppress the proliferation of effector T cells in a contact-dependent, cytokine-independent manner. In contrast, other types of TReg cells can be induced from naive CD4 cells in the periphery, such as IL-10-producing TR1 cells and TGF-β-producing Th3 cells (Groux et al., 1997; O’Garra et al., 2004; Grazia-Roncarolo et al., 2006; Nishikawa and Sakaguchi, 2010). Such “induced” CD4^+^CD25^−^ TReg subpopulations (iTReg) exert suppression mostly through soluble factors and their suppressive function is not strictly associated with a high level of FoxP3 expression. Moreover, human TReg cell subpopulations have also been further divided into two subsets based on their expression of the “resting” CD45RA (a marker of naïve or antigen-inexperienced cells) or “activated” CD45RO (a marker for memory or antigen-experienced T cells) cell surface markers (Vukmanovic-Stejic et al., 2006; Miyara et al., 2009; Miyara and Sakaguchi, 2011; Duhen et al., 2012) further suggesting different levels of activation and/or differentiation among these CD4 subsets. More recently, another inducible subpopulation of the CD4^+^ TReg cell subset have been reported in both human and murine systems that involve production of IL-35 and are thus referred to as iTreg35 cells (Collison et al., 2010; Chaturvedi et al., 2011). Notably, these cells are phenotypically and functionally distinct from other subpopulations of TReg cells described thus far in that they do not express FoxP3 and they mediate immunosuppression via IL-35 and seemingly independent of IL-10, TGF-β, the immunomodulatory receptor CTLA-4, or any other currently known TReg cell-associated suppressive molecule. Although it seems that human nTReg cells do not express IL-35 (Bardel et al., 2008), naïve human CD4 T cells can be induced to develop into iTReg35 cells in the presence of IL-35 or activated DCs (Collison et al., 2010; Seyerl et al., 2010). Alternatively, it has been suggested that human TReg subpopulations can be further classified by their expression of select chemokine receptors that correspond to Th cell lineage-specific immune responses (Duhen et al., 2012). For example, TReg subpopulations co-expressing CCR6 (Th17-associated responses), CXCR3 (Th1-associated responses), CCR4 (Th2-associated responses), and CCR10 (Th22-associated responses) enable human TReg cell subpopulations with unique specificities and immunomodulatory functions to target defined immune environments during different types of inflammatory responses so as to exert an “appropriate” regulatory process. Thus, suggesting that Th and TReg cells undergo functional specialization in parallel, resulting in the development of TReg cell subpopulations capable of co-localizing and effectively regulating different types of Th cell responses *in vivo* (Hall et al., 2011; Duhen et al., 2012). In any instance, the precise mechanisms by which these various subpopulations of TReg cells function to maintain the balance between protective tumor immunity and establishing or rebalancing immune cell homeostasis remains poorly understood. However, several mechanisms responsible for preventing inflammatory disease by restraining aberrant responses to self or innocuous antigens have been identified (Vignali et al., 2008; Shevach, 2009; Qureshi et al., 2011; Yamaguchi et al., 2011; Vignali, 2012; Wing and Sakaguchi, 2012). These include both cell contact and soluble factor-dependent mechanisms, such as production of IL-10; the production and surface expression of TGF-β; the production of IL-35; the release of cytolytic molecules such as granzyme and perforin; the consumption of IL-2 through the high density expression of cell surface CD25 (the alpha chain of the IL-2 receptor) which weans effector T cells from IL-2; the degradation of ATP through ectonucleotidases; and expression of the inhibitory receptors CTLA-4, which outcompetes receptor CD28 on effector T cells for access to the co-stimulatory molecules CD80 and CD86 on APCs.

## CD4 Memory T Cell Development and Their Role in the Antitumor Response

During the antitumor response a small population of tumor-specific CD4 effector T cells may develop into memory T cells that retain their previous effector functions and rapidly produce effector cytokines (McKinstry et al., 2010; Taylor and Jenkins, 2011; Strutt et al., 2012). Their ability to remember previously encountered antigens leads to faster responses to tumor antigen re-exposure and thus may play a role in preventing disease relapse in cancer patients. Alternatively, as discussed earlier, it can shape tumor cell immunogenicity and modulate immune response dynamics to influence disease progression (Schreiber et al., 2011; Vesely et al., 2011). In humans, different isoforms of the CD45 molecule are often used to differentiate naïve and memory cells with the former expressing CD45RA and the latter expressing CD45RO (Ahmed and Gray, 1996). Increased expression of other surface molecules such as the CD95 death receptor has also been shown to differentiate memory cells from naïve CD4 T cells. Moreover, memory T cells have been divided into two general subgroups based on their patterns of migration (Sallusto et al., 1999). Central memory T cells (T_CM_) express the CC-chemokine receptor CCR7 and L-selectin CD62L following activation. Expression of these receptors enable the T_CM_ subgroup to recirculate through secondary lymphoid organs such as lymph nodes. Such circulation is beneficial since DCs from diverse tissue sites continuously bring antigen to the draining lymph nodes, thereby increasing the effective area of memory cell immunosurveillance for progressing tumor growth due to metastases and/or occult cell outgrowth. Alternatively, effector memory T cells (T_EM_) lack expression of CCR7 or CD62L and thus have a propensity to migrate to peripheral tissues in response to localized inflammatory stimuli and bolster the process of immune surveillance at such sites. Moreover, expression of other surface chemokine receptors such as CCR5 have been associated with polarized Th1 memory T cell subsets (Loetscher et al., 1998; Sallusto et al., 1998; Kim et al., 2001; Luther and Cyster, 2001; Charo and Ransohoff, 2006). In either instance, retention and tissue tropism of memory CD4 T cell subsets and their diverse functional capacities are dependent, in part, on specific interactions between adhesion molecules and effector cell chemokine receptors that induce T cell subset localization and influence tumor environments. This has been shown in studies with cancer patients where increased intratumoral memory T cell levels were associated with longer disease free and overall survival rates (Pagès et al., 2005; Bindea et al., 2010). In another study involving colon cancer patients, histopathological analysis showed the presence of “patches” of T_EM_ cells located within either the center or invasive margins of the tumor that further correlated with good clinical outcome (Galon et al., 2006). These investigators suggested that memory cell localization at select regions within the tumor mass may be associated with not only enhanced antitumor immune responses but also effective control of metastatic escape. However, as briefly mentioned earlier, such observations and effective antitumor responses may not only depend on memory cell phenotype and localization, but also their functional memory precursor phenotype and ability for “cellular plasticity” within the hostile tumor environment.

In addition to surface markers, differential expression levels of select transcription factors have been associated with promoting effector and memory T cell development. In studies using various genetically modified mouse strains, investigators have shown that under conditions of inflammation, elevated levels of the transcription factor T-bet (*Tbx21*) among responding CD8 T cells promoted the generation of terminally differentiated short-lived effector cells whereas lower levels facilitated long-lived, self renewing memory T cell development (Joshi et al., 2005; Lazarevic and Glimcher, 2011). In more recent studies, it was shown that IL-12 augmented activity of the kinase mammalian target of ramamycin (mTOR) (Rao et al., 2010; Cox et al., 2011) which is essential for sustained T-bet expression and the generation of effector CD8 T cells. Subsequently, inhibition of mTOR activity blocked T-bet expression and promoted elevated and sustained levels of the closely related T-bet transcription factor eomesodermin (*Eomes*) that is associated with the development of memory T cells (Pearce et al., 2003; Intlekofer et al., 2005, 2008; Joshi et al., 2005; Rao et al., 2010). Moreover, over-expression of eomesodermin or T-bet has been shown to be sufficient to induce expression of IFN-γ, perforin, and granzyme B in CD8 T cells (Pearce et al., 2003; Cruz-Guilloty et al., 2009). Thus suggesting that (i) the transcription factors T-bet and eomesodermin have cooperative and partially redundant functions in CD8 T cell differentiation and fate (Rao et al., 2010) and (ii) the balance between the two transcription factors, as “instructed” by mTOR kinase activity, can determine the CD8 effector cell fate verses memory cell fate (Araki et al., 2009; Rao et al., 2010). Although the transition of CD4 effector to memory T cell phenotype is less defined, evidence using a murine viral infection model has shown a similar correlation with decreased T-bet (Tbx21) expression levels and potential Th1 memory cell development (Marshall et al., 2011). Furthermore, in a mouse model of allergic airway inflammation, IL-5 production among a Th2 memory cell subpopulation was shown to be uniquely regulated by the expression of eomesodermin (*Eomes*) suggesting a role for this transcription factor in the regulation of polarized CD4 T cell functions (Endo et al., 2011). Whereas, in a study using peripheral blood from healthy human donors, expression of T-bet was shown to be up-regulated among specific Th1 memory cell subpopulations following TCR stimulation whereas elevated expression levels of eomesodermin (*Eomes*) were associated with a higher level of IFN-γ production during the recall response in a corresponding cell subpopulation (Narayanan et al., 2010). Lastly, similar results were observed in the murine system suggesting a role for Eomesodermin (*Eomes*) in the development of Th1 cell differentiation and memory phenotype under various stimulating conditions *ex vivo* (Suto et al., 2006; Yang et al., 2008; Hirschhorn-Cymerman et al., 2012). Collectively, it is unclear which responding CD4 effector T cells make the transition to a memory phenotype, but these recent studies suggest that differential expression levels and balance between transcription factors promote and/or facilitate the T cells potential to do so. In either instance, this “phenotypic progression” from effector to memory T cell provides a qualitative advantage in the antigen-specific antitumor response by enhancing immune response time, the need for less co-simulation and more vigorous proliferation especially at lower levels of tumor antigen exposure when compared to that of antigen-inexperienced T cells.

## Helper Functions and the Potentially “Good” and “Bad” Sides of CD4 Effector T Cell Subsets in the Antitumor Immune Response

Although the best studied pathways of CD4 T cell-mediated help are those that promote antibody production by B cells, such cells also enhance tumor-specific CD8 T cell responses during disease progression and contribute to the maintenance of a functional memory CD8 T cell pool (Pardoll and Topalian, 1998). Various CD4 T cell subsets can also alter the function of APCs (especially DCs) and innate immune cells (Hung et al., 1998). In addition to enhancing and/or regulating T cell-mediated responses, that include both promoting long-term immunity and establishing or rebalancing immune cell homeostasis, CD4 T cells can also have a direct role in tumor elimination (Pardoll and Topalian, 1998; Quezada et al., 2010; Xie et al., 2010). Paradoxically, several experimental and clinical observations have recently shown that such CD4 effector cell subsets and their signature cytokines can not only contribute to effective antitumor responses but also facilitate tumor-promoting activities. In this section of the review, we will discuss these points and focus on the three most studied and potentially most promising CD4 effector T cell subsets involved in antitumor immunity and therapy, namely the Th1, Th17, and TReg cell subsets.

### The Th1 and IFN-γ Paradox

Th1 cells are potent inducers of cell-mediated immunity and inflammation. Through studies using various murine tumor models, Th1-mediated immune responses have been shown to participate and facilitate in the elimination of established tumors and reduce tumor development and susceptibility to carcinogenesis. Moreover, it has been observed in studies of patients with various cancer types that favorable clinical outcomes, as assessed by disease free and overall survival, can be attributed to an enhanced and coordinated Th1 effector cell infiltration within the tumors of these patients (Fridman et al., 2011). IFN-γ is produced predominantly by the Th1 CD4 effector T cell subset. Tumor antigen-specific Th1 cells control tumors, in part, through the secretion of IFN-γ that can have both direct and indirect effects on immune activation and modulation (Mumberg et al., 1999; Zaidi and Merlino, 2011). IFN-γ derived from Th1 cells can induce a cascade of events involving the priming and maturation of cytolytic CD8 T cells through activation of DCs at the sites of tumor growth and further induce tumor elimination through activation of NK cells and type 1 macrophages (Corthay et al., 2005; Quezada et al., 2010; Palucka and Banchereau, 2012). Moreover, IFN-γ can induce development of the Th1 cell lineage, rather than the potentially tumor-promoting Th2 lineage, and further promote expression of the chemokine receptor CXCR3 and its ligands CXCL9, CXCL10, and CXCL11 that can specifically attract and enhance Th1 cell localization to sites of tumor growth and inflammation (Rotondi et al., 2003). Other studies have suggested that IFN-γ actually inhibits the generation and/or activation of naturally occurring TReg cell subsets (Nishikawa et al., 2005; Caretto et al., 2010). Similarly, another group showed IFN-γ signaling caused cell cycle arrest in TReg cells suggesting that this IFN-γ-dependent mechanism could counteract the ability of TReg cells to protect tumors in cancer patients (Cao et al., 2009). Aside from its immune stimulatory roles and affects on various T cell subpopulation dynamics, IFN-γ can up-regulate HLA class I and class II molecules on tumor cell populations that aid in facilitating cytolytic T cell recognition and elimination of tumors. Studies in both human and murine systems have shown IFN-γ to inhibit cancer cell proliferation (Bromberg et al., 1996; Chin et al., 1996; Hobeika et al., 1999; Platanias et al., 1999; Zaidi and Merlino, 2011), promote tumor cell apoptosis through effects on the expression of caspases, FAS (also known as CD95), and TRAIL (Takeda et al., 2002; Chin et al., 1997; Xu et al., 1998; Meng and El-Deiry, 2001), and inhibit angiogenesis within the tumor environment (Luster and Leder, 1993; Coughlin et al., 1998; Ruegg et al., 1998. Beatty and Paterson, 2001). With respect to angiogenesis, IFN-γ is a potent inducer of several angiostatic chemokines, such as CXCL9 and CXCL10, from a variety of cells, including monocytes, macrophages, fibroblasts, endothelial cells, and tumor cells (Luster and Ravetch, 1987; Farber, 1990; Arenberg et al., 1996; Cole et al., 1998). This may contribute to a shift the local biologic balance between angiogenic and angiostatic chemokines that results in anti-angiogenesis and tumor-associated vascular inhibition. Of course, the different IFN-γ-inducible processes and effects, that are responsible for directly limiting tumor growth and progression, are not only dependent on tumor type, but also cytokine concentration and expression of the extracellular domains of the IFN-γ receptor subunits and their intracellular signaling transmission pathways among the various cells within a dynamic tumor environment (Ealick et al., 1991; Boehm et al., 1997).

Alternatively, a dual role for IFN-γ in the context of malignancy has been reported and associated with contributing to enhanced tumor growth and metastases (Dunn et al., 2006; Schreiber et al., 2011). Several reports have suggested that IFN-γ, possibly derived from Th1 cells, can up-regulate the surface expression of the immunoinhibitory molecule B7-H1 on tumor-associated APCs (Dong et al., 2002; Liu et al., 2007; Zou and Chen, 2008; Wu et al., 2009a; Kondo et al., 2010). Under such conditions, cross-talk between these APCs and T cell expressing the corresponding ligand, PD-1, could result in a coordinated suppressive and tolerogenic environment. In addition, it has been shown that T cell-derived IFN-γ can interact with tumor cells and tumor-associated APC to induce the expression of indoleamine 2,3-dioxygenase (IDO), an enzyme that degrades the essential amino acid tryptophan that leads to the suppression of T cell immunity (Carlin et al., 1987; Munn et al., 2002; Fallarino et al., 2003; Zou, 2005; Sharma et al., 2007; Mellor and Munn, 2008; Muller et al., 2008). It has been shown that IFN-γ can enhance the presence of myeloid-derived suppressor cells (MDSC), in an IFN-γ-dependent manner, within the tumor microenvironment resulting in the suppression of T cell responses (Ostrand-Rosenberg and Sinha, 2009; Gabrilovich et al., 2012). Lastly, it has been reported that IFN-γ can facilitate and/or mediate either contraction of the CD4 T cell population via induction of apoptosis (Berner et al., 2007) or up-regulate and induce the development of TReg cells (Agnello et al., 2003; Liu et al., 2009; Campbell and Koch, 2011). Such IFN-γ-mediated activity derived from Th1 cells infiltrating sites of tumor growth can conceivably undermine the antitumor immune response by negatively affecting T cell population dynamics *in vivo*. For example, Th1 cells may possess homeostatic functions under select conditions within the tumor that can influence the generation, survival and balance of CD4 and CD8 effector and memory T cell subpopulation pools necessary for effective antitumor responses. Consequently, such immune-mediated effects on the local tumor environment could be responsible for the promotion of tumor cell dormancy and contribute to the maintenance, potential progression and/or re-emergence of occult tumor cells or cancer-related stem cells (Mellor and Munn, 2008; Schreiber et al., 2011).

Finally, with regards to clinical application, recombinant IFN-γ was initially used to treat chronic myelogenous leukemia, alone and in combination with IFN-α, but failed to show any significant positive outcome (Kurzrock et al., 1987; Kloke et al., 1992). Since then, recombinant IFN-γ has been used in the clinical management of a variety of malignancies including bladder carcinoma, colorectal cancer, ovarian cancer, and adult T cell leukemia; however, the results have been mixed (Miller et al., 2009). In melanoma patients, early small scale clinical trials were largely inconclusive (Creagan et al., 1987; Ernstoff et al., 1987; Kowalzick et al., 1990; Kopp et al., 1993). In another trial for adjuvant application in patients with melanoma, studies were prematurely terminated due to the observations that IFN-γ-treated patients fared worse than the untreated population (Meyskens et al., 1990, 1995). Although the clinical application and therapeutic effects of directly administered recombinant IFN-γ in cancer patients appears marginal, there is limited experience in investigations focused on therapies utilizing direct transfer of tumor-reactive CD4 T cells secreting IFN-γ. Several earlier clinical studies utilizing various T cell transfer therapies have suggested that the incorporation of CD4 T cells would heighten therapeutic efficacy and improve clinical outcome (Walter et al., 1995; Dudley et al., 2002; Ho et al., 2002; Levine et al., 2002; Kershaw et al., 2006; Bollard et al., 2007). One of the initial clinical studies showing autologous IFN-γ producing CD4 T cell transfer as an effective therapeutic agent in cancer patients was performed by Hunder et al. Using an autologous CD4 T cell clone with specificity to the melanoma-associated antigen NY-ESO-1, these investigators showed that transferred tumor-reactive IFN-γ-producing CD4 T cells mediated a durable clinical remission and promoted endogenous responses against melanoma antigens other than NY-ESO-1 in a melanoma patient (Hunder et al., 2008). Furthermore, the patient experienced a complete response with persistence of the transferred cells and a concomitant induction of melanoma antigen-reactive CD8 T cells. In our adoptive T cell therapy studies with late stage ovarian cancer patients, using peripheral blood-derived MUC1 peptide-stimulated Th1-like effector T cells, we reported that autologous T cell re-stimulation and subsequent intra-peritoneal re-infusion modulated endogenous T cell-mediated immune responses and systemic T cell subpopulation dynamics that were associated with enhanced patient survival (Dobrzanski et al., 2011). In spite of the limited numbers of such studies investigating CD4 T cell based immunotherapy in cancer patients, it is becoming apparent that Th1 cells possess the capacity to modulate the immune response and potentially enhance tumor immunity in the clinical setting.

### The immunomodulation paradox of IL-10 derived from CD4 effector T cells

Several studies support the view that IL-10 may diminish the immune response against cancer by directly inhibiting cell activation of select human T cell subpopulations (de Waal Malefyt et al., 1993; Taga et al., 1993; Joss et al., 2000). IL-10 can also act as a negative mediator in the cross-talk between innate and adaptive antitumor immunity. For example, it has been reported that the IL-10 immunosuppressive activity on T cells is mainly indirect and is functionally linked to other immune cells such as TReg cells, MDSC, and APCs. In the case of APCs, IL-10 restrains antigen presentation via its inhibition of MHC and co-stimulatory B7 family member molecules (Vicari and Trinchieri, 2004; O’Garra and Murphy, 2009), stimulates up-regulation of inhibitory B7 family members (Curiel et al., 2003; Kryczek et al., 2006; Zou and Chen, 2008), down-regulates IL-12 production, and inhibits DC maturation and differentiation (Moore et al., 2001). *In vitro* studies have shown that T cells can be anergized toward melanoma-associated antigens when stimulated with IL-10 conditioned DCs (Steinbrink et al., 1999), and DCs which infiltrate progressing melanoma metastases in humans, have been characterized to express low levels of CD86 and IL-12 but possess an enhanced capacity to produce IL-10 (Enk et al., 1997). Moreover, IL-10 producing monocytes and select populations of the myeloid lineage, which inhibit T cell proliferation, have been isolated from the ascites of patients with ovarian carcinomas (Loercher et al., 1999). These cells and their subpopulations, such as MDSCs, can promote the local clonal expansion and/or induce conversion of naïve CD4 T cells into TReg cell populations (Gabrilovich et al., 2012). Moreover, it has been reported that the CD4^+^ Tr1 regulatory cells produce antigen-driven IL-10 that is responsible for peripheral immune tolerance through the impaired activation and regulation of CTL, Th1, and other effector Th cell subsets that further facilitate elevated tumor growth through immune escape mechanisms (Seo et al., 2002). It is now clear that IL-10 can not only mediates inducible TReg cell immunosuppressive activity but also plays a direct role in their genesis (Roncarolo et al., 2001). *In vitro* studies have shown that IL-10 treatment can convert different types of tumor cells, such as melanoma and lymphoma, to a CTL-resistant phenotype by decreasing the expression of HLA class I molecules on their surface (Petersson et al., 1998; Kurte et al., 2004). Similarly, IL-10 production by human basal and squamous cell carcinoma prevents *in vitro* lysis of autologous malignant cells by cytolytic T lymphocytes (Kim et al., 1995). Lastly, recent studies have shown that endogenous IL-10 can potentially limit the pro-tumor and/or antitumor effects of Th17-mediated inflammation either indirectly by promoting the regulatory functions of both FoxP3^+^ and FoxP3^−^ cells or directly by interacting with IL-10 receptors on Th17 cells in an IL-10 signaling-dependent manner (Chaudhry et al., 2011; Huber et al., 2011). Collectively these studies in both the animal and human systems suggests that IL-10 is involved in both direct and indirect tumor immunosuppressive networks that can promote and facilitate tumor immune tolerance resulting in malignant progression.

Although IL-10 is generally regarded as an anti-inflammatory and immunosuppressive cytokine that favors tumor escape from immune surveillance, evidence is accumulating that IL-10 also possesses immunomodulatory properties that support antitumor immunity. For example, transfection of tumor cells with IL-10 or systemic administration of exogenous IL-10 significantly suppressed tumor growth and led to tumor rejection in several different murine tumor models *in vivo* (Giovarelli et al., 1995; Berman et al., 1996; Fujii et al., 2001; Mumm et al., 2011). Moreover, such antitumor effects of IL-10 were dependent on CD8 T cells. *In vitro* studies have further shown that IL-10 can induce proliferation and cytotoxic activity of human CD8 T cells and function as a chemoattractant for CD8 T cells (Chen and Zlotnik, 1991; Jinquan et al., 1993; Groux et al., 1998; Santin et al., 2000). In more recent studies using IL-10 and IL-10 receptor knockout and transgenic mouse strains, investigators reported that IL-10 directly mediated intratumoral activation and expansion of resident tumor-reactive CD8 T cells that independently rejected established tumor growth and progression (Emmerich et al., 2012). Alternatively, another group using IL-10 knockout mice showed that IL-10 indirectly hindered tumor development, growth, and progression by impeding the development of both MDSCs and CD4^+^ TReg cells which presumably contributed to immune suppression, carcinogenesis, and tumor pathology (Tanikawa et al., 2012). Lastly, recombinant IL-10 has been associated with stimulating pro-inflammatory responses, such as IFN-γ production, when administered to humans (Lauw et al., 2000; Tao et al., 2001; Tilg et al., 2002). Since it is clear that nearly all CD4 effector T cell subsets can potentially produce IL-10, it is conceivable that endogenous IL-10 (such as IL-10 derived from CD4 helper/effector T cells infiltrating sites of tumor growth) may exhibits both antitumor and pro-tumor activities (Mocellin et al., 2001). Under both scenarios, IL-10 may influence tumor cells through the development, recruitment, and/or activation of various immune response cells, including tumor-reactive CD8 and other CD4 effector T cell subsets. Alternatively, a body of both clinical and pre-clinical data is emerging showing that IL-10 can influence tumor growth and progression by non-immune-related phenomena such as the inhibition of angiogenesis and induction of tumor cell apoptosis (Mocellin et al., 2005). In either instance, the role of endogenous IL-10 as a mediator of either tumor escape or successful immune surveillance may depend upon the conditions of initial carcinogenesis and tumor type, level of tumor progression, and the presence of responding immune cell populations at the sites of tumor growth.

Many CD4 Th cell subsets can potentially produce IL-10, as well as their hallmark cytokines, following engagement of their TCR with antigen (Sariava and O’Gara, 2010). The presence of reactive tumor infiltrating CD4 helper T cells have been associated with good clinical outcomes in patients with select cancer types (Pagès et al., 2005). Interestingly, this broaches the possibility that IL-10-derived from such Th cells may act, in part, as an immunological adjuvant in the antitumor response to cancer. A subset of Th1 cells has been identified in both murine and human systems and found to either stably or transiently produce both IL-10 and IFN-γ during periods of chronic inflammation and disease (Jankovic et al., 2010; Cope et al., 2011). Several signals have been found to stimulate the generation of such dual cytokine-expressing effector cell subpopulations, including high levels of antigen, soluble factors such as IL-12 and IL-27, and co-stimulatory signals such as ICOS (Jankovic et al., 2010). More recently, it has been suggested that such cytokine switching and/or co-expression in both human and mouse cells of the Th1 lineage may be linked to the role of the complement regulator and T cell co-stimulatory molecule, CD46 with the addition of either TCR engagement or high amounts of IL-12 (Meyaard et al., 1996; Cardone et al., 2010). In either instance, it has been suggested that Th1 effector cell responses are auto-regulated through not only extrinsic but also intrinsic negative feedback loops via the co-induction of IL-10 and IFN-γ. Thus, it is conceivable that the relative amounts and/or duration of IFN-γ and IL-10 produced by such double-positive cytokine secreting effector cell subsets might define the antitumor and/or inflammatory immune response within the tumor microenvironment that results in either tumor eradication or tolerance induction and disease progression. In our recent studies investigating the therapeutic role of adoptively transferred Th1-like effector cells in patients with ovarian cancer, we reported that autologous IFN-γ-secreting CD4 effector cells used in the treatment of long-term surviving patients co-produced higher levels of CD4 effector cell-derived IL-10 when compared to that of short-term survivors. We suggest that such heightened or variable levels of effector cell-derived IL-10, either in combination with IFN-γ or alone, may contribute, in part, to enhancing patient antitumor responses by modulating select effector T cell subsets, such as CD4^+^ TReg cells and their subpopulations. Conceivably, such modulation in effector cell population dynamics could affect the balance between effective and ineffective antitumor responses and patient survival. Although the molecular mechanisms and roles underlying the effects of IL-10 have not been well characterized, the biological activities of IL-10 in tumor immunity and pathology appear highly context-dependent.

### The Th17 and IL-17 paradox

IL-17 secreting Th cells (Th17) have been implicated in promoting inflammation responsible for immunopathology in both cancer and several autoimmune disorders. Studies in various murine tumor models have suggested that Th17 cells may be associated with tumor initiation and growth in the context of chronic inflammation (Kawakami et al., 2009; Wang et al., 2009; Wu et al., 2009b). In patients with hormone-resistant prostate cancer, an inverse correlation has been reported between pretreatment circulating levels of Th17 cell numbers and time to disease progression suggesting that Th17 cells may accelerate tumor development in such patients (Derhovanessian et al., 2009). Alternatively, others have suggested that Th17 cells may contribute to protective antitumor responses in select human malignancies whereas Th17-associated cytokines may be the contributing factors related to tumor initiation and growth. In studies utilizing various genetically modified murine tumor models, investigators have shown that endogenous IL-17 (such as IL-17 derived from Th17 cells infiltrating sites of tumor growth) could promote tumor growth by inducing tumor vascularization, suggesting that the cellular targets of IL-17 in the tumor microenvironment can be vascular endothelial cells, stromal cells, and cells of the tumor itself (Numasaki et al., 2003; Wilke et al., 2011). Later studies showed that IL-17 induced a wide range of angiogenic mediators (Numasaki et al., 2004; Takahashi et al., 2005; Honorati et al., 2006), including vascular endothelial growth factor (VGEF), that markedly promotes inflammation and tumor angiogenesis. Alternatively, IL-17 has been shown to induce IL-6 production from tumor cells and tumor-associated stromal cells, which in turn activate STAT-3, an oncogenic transcription factor that upregulates pro-survival and pro-angiogenic gene levels in transformed cells (Wang et al., 2009). Furthermore, IL-17 has been shown to selectively enhance the production of angiogenic chemokines such as CXCL1, CXCL5, CXCL6, and CXCL8 in tumor cells and epithelial cells (Numasaki et al., 2005; Lee et al., 2008). Thus, the biological activities and tumor-promoting effects of endogenous IL-17-mediated inflammatory responses appear to be dependent on differences in local cytokine concentrations, bioavailability, and presence of select responding target tissues. Moreover, IL-17 appears highly context-dependent with respect to tumor type, stages of development, and host immune status as cytokine-mediated effects have been shown to be heightened in immunocompromised animals (Murugaiyan and Saha, 2009).

Alternatively, pre-clinical murine tumor studies have correlated the presence of intratumoral Th17 cells with reduced tumor growth and effective antitumor immunity (Muranski et al., 2008; Martin-Orozco et al., 2009). Polarized Th17 cells have been observed in distinct human cancer types, including colon, melanoma, pancreatic, hepatocellular, and ovarian (Kryczek et al., 2009a). In clinical studies, patients with advanced ovarian cancer were observed to possess elevated levels of both intratumoral Th17 cell numbers and IL-17 concentrations within patient ascites that correlated with improved survival (Kryczek et al., 2009a; Wilke et al., 2011). Similar results have been observed in patients with other malignancies suggesting a beneficial role for Th17 cells in cancer (Sfanos et al., 2008; Ye et al., 2010; Chen et al., 2011). Moreover, Th17 cells have been observed to be negatively correlated with TReg cells and positively correlated with effector immune cells including IFN-γ-secreting Th1 cells, cytotoxic CD8 T cells, and NK cells within the tumor microenvironment (Kryczek et al., 2009a; Zou and Restifo, 2010; Wilke et al., 2011). However, their roles and mechanisms of action in the antitumor response are not well understood. It has been suggested that possible protective mechanism(s) mediated by Th17 cells include their capacity to secrete multiple and functionally distinct cytokines such as IL-17A, IL-17F, IL-22, and IL-21. For example, IL-21 production has been shown to sustain CD8 T cell responses (Moroz et al., 2004; Zeng et al., 2005; Frederiksen et al., 2008). Moreover, Th17 cells have been shown to produce the chemokine CCL20 which can promote DC trafficking to the sites of tumor growth in a CCL20-CCR6 dependent manner. Recruitment of such DCs can effectively result in the priming and activation of CD8 T cells that result in enhanced CTL activity. In addition, IL-17 has been shown to promote maturation of DC progenitors (Antonysamy et al., 1999) and induce IL-12 production from macrophages (Jovanovic et al., 1998). Thus, these studies suggest that the combined cellular products from both Th17 and additional immune cells infiltrating the tumor, and the interaction between these cell types, may play a role in the balance between effective antitumor immunity and pro-tumor responses.

Investigations on the association between Th17 cells and promising clinical outcomes in cancer patients, have suggested a link and close interplay between the Th17 and Th1 cell lineages (Kryczek et al., 2009a; Marshall et al., 2012). Within this positive association between intratumoral Th17 and IFN-γ-secreting Th1 cells, human Th17 cell populations producing both IL-17 and IFN-γ have been identified (Annunziato et al., 2007; Hamaï et al., 2012). It has been reported that these dual cytokine secreting cell populations are exclusively derived from Th17 cells and not initially differentiated Th1 cells (Hirota et al., 2011). These “converted” and/or “re-differentiated” Th17 cells express the Th1 related transcription factor T-bet in addition to the IL-17-related transcription factor ROR-γt (Annunziato et al., 2007). Stimulation by IL-12 rapidly down-regulated IL-17 production and induced expression of IFN-γ through enhanced T-bet expression and the subsequent down-regulation of ROR-γt expression (Annunziato et al., 2007; Annunziato and Romagnani, 2010; Lazarevic et al., 2011). Moreover, this shift in phenotype appeared to be facilitated by the low but constitutive expression of IL-12Rβ2 among Th17 cells (Annunziato et al., 2007; Lee et al., 2009). Thus these findings provided a molecular basis to explain Th17 cell plasticity and/or conversion to the Th1 cell lineage and further supports the concept that such events can occur within portions of the Th17 cell population under inflammatory conditions such as that of a hostile tumor environment *in vivo* (Muranski et al., 2008; Bending et al., 2009; Martin-Orozco et al., 2009b; Annunziato and Romagnani, 2010; Murphy and Stockinger, 2010; Nistala et al., 2010; Hamaï et al., 2012; Marshall et al., 2012). This process of Th17 cellular plasticity appears highly context-dependent and can be influenced, in part, by the cytokine milieu produced by innate immune cells within the inflammatory environment. Consequently, such conversion of the Th17 cell population into the Th1 cell lineage, can have important biological implications in tumor immunity and disease progression. As mentioned earlier, both effector cell-derived IL-17 and IFN-γ can potentially promote or suppress the generation of effective immune responses through a myriad of different mechanisms (Xiao et al., 2009; Tosolini et al., 2011). Although the role of Th17 cells co-producing IL-17 and IFN-γ is not clear, it has been suggested that both cytokines can either synergistically or independently induce the production of functionally diverse chemokines within the tumor environment which in turn can recruit and promote distinct types of effector T cells and/or other immune cells that can influence antitumor immune responses and mediate tumor regression or progression (Kryczek et al., 2009b; Martin-Orozco et al., 2009; Kesselring et al., 2010). Thus, it is conceivable that the relative quantity and/or duration of either IL-17 or IFN-γ produced by such double-positive cytokine secreting Th17 cell subpopulations may define the antitumor immune response. Moreover, the type of tumor, the cells within its microenvironment, and their responsiveness to the various tumor-associated cytokines may further promote and influence an imbalance between pro-tumor verses antitumor effects. For example, recent studies in the murine system by Huber et al., have shown that both IL-17- and IL-17/IFN-γ-producing Th17 cells express higher surface levels of the IL-10Rα when compared to that Th1 cells and that the potential antitumor or pro-tumor effects of the Th17-mediated inflammatory response can be more readily suppressed by endogenously produced IL-10 (Huber et al., 2011). Since many lineage-related tumor types can initially possess and generally favor similar microenvironments that can induce and selectively affect specific effector T cell-mediated responses, this may partially explain why Th17 cells have been observed and associated with protective tumor immunity in only some cancers but not all (Kryczek et al., 2009a). The pro-tumor verses antitumor effects of such Th17 effector cell subpopulations may thus represent a “balance” between IL-17 and IFN-γ cytokine production that can facilitate either tumor promotion or regression. Further identification and characterization of the mechanisms involved in the induction of tumor-reactive Th17 effector cells and their activities in cancer patients may offer significant advantages for future treatment strategies of human malignancies.

### CD4^+^ TReg cell subpopulations in immune regulation and the antitumor response

The immunoregulatory roles of CD4^+^ TReg cell subsets have been associated with the prevention of immunopathology during excessive and/or unwanted inflammation and prevention of autoimmune disease. However, in the context of cancer, such cells have been associated with facilitating the suppression of the antitumor response through various tolerance induction and tissue homeostatic mechanisms. However, the role and prognostic value of TReg cells in cancer has recently been disputed (Wilke et al., 2010; Tosolini et al., 2011; deLeeuw et al., 2012). It has initially been reported that high TReg cell frequencies infiltrating the tumor environment correlate with more advanced disease and poor prognosis in patients. In ovarian, pancreatic, and breast cancer patients, either systemic or local FoxP3^+^ TReg cell expression has been associated with both a poor prognosis and diminished survival rates (Woo et al., 2001; Liyanage et al., 2002; Wolf et al., 2003, 2005; Curiel et al., 2004; Li et al., 2005; Bates et al., 2006; Merlo et al., 2009). Curiel and colleagues reported that the presence of high numbers of CD4^+^FoxP3^+^ T cells in malignant ascites of patients with ovarian carcinomas correlated with advanced tumor staging and reduced survival. Alternatively, in colorectal cancer, several investigators did not find any differences between patients with high or low TReg cell infiltration (Loddenkemper et al., 2006) whereas others have found an improved survival associated with a high density of local and systemic FoxP3^+^ cells suggesting no major immunosuppressive role of TReg cells in colorectal cancer (Salama et al., 2009). Moreover, it has been suggested that the presence and levels of various TReg cell subsets in cancer patients may be beneficial to survival (Alvaro et al., 2005; Erdman et al., 2005; Grivennikov et al., 2010; Wilke et al., 2010; deLeeuw et al., 2012). None-the-less, in early clinical studies investigating adoptive T cell transfer therapies in patients with select cancer types, it was observed that TReg cell depletion prior to therapy can enhance clinically relevant immune responses to such treatments (Muranski et al., 2006; Wrzesinski et al., 2007; Dudley et al., 2008; Porter et al., 2011; Rosenberg et al., 2011; Le and Jaffee, 2012; Yao et al., 2012). These findings fit with the general notion that TReg cells suppress adaptive immune responses and led many groups to pursue various cytoablative strategies to deplete such cells from cancer patients receiving immunotherapy as a means to enhance clinical responses. In contrast, others have observed the induction of effective antitumor responses following administration of various immunotherapeutic strategies in the absence of cytoablative treatments, and have suggested that such responses are likely due to the balance between effector T cells (i.e., either CD4 or CD8) and TReg cells within treated cancer patients (Alvaro et al., 2005; Quezada et al., 2006; Hunder et al., 2008; Le and Jaffee, 2012; Liu et al., 2012a). Along these lines, in our clinical study investigating adoptive T cell therapy using autologous Th1-like effector cells in the treatment of ovarian cancer patients, we observed enhanced T cell-mediated immune responses in long-term surviving patients that appeared to correlate with differences in their ratios of “inducible” verses “natural” TReg cell subpopulations when compared to that of short-term survivors receiving similar treatments (Dobrzanski et al., 2009, 2011). We suggest that such patient responses did not appear to be dependent on TReg cell numbers but upon their subpopulation ratios within responding patients. Although the precise mechanisms by which these regulatory cells and their various subpopulations (and those that have yet to be defined) potentially function to establish or rebalance immune cell homeostasis and sustain the “balance” between tumor immunity, suppression, and tolerance remains poorly understood, it could be speculated that a collaboration and cross-talk among these various TReg subpopulations are required for the maintenance and control of effective immune responses. Thus, a conceivable role for co-therapeutic approaches targeting modulation, and not depletion, of the TReg cellular network in patients with select tumor types may be an alternative and potentially effective therapeutic approach to treating cancer patients.

Select chemokines and their corresponding receptors have been shown to play a role in the recruitment of specific T cell subsets into tumors and sites of inflammation (Sallusto et al., 1997, 1998; Bonecchi et al., 1998; Loetscher et al., 1998; Hirai et al., 2001; Iellem et al., 2001; Muthuswamy et al., 2012). Among human TReg cells, the chemokine receptor CCR4, and its ligands CCL22 and CCL17, are believed to be the most predominant chemokine-related mechanism responsible for TReg cell trafficking to tumors (Iellem et al., 2001). It has been reported that production of the chemokine CCL22 is associated with human ovarian cancer (Iellem et al., 2001; Curiel et al., 2004) and has also been observed in other types of malignancies, such as gastric cancer (Haas et al., 2008, 2009), Hodgkin’s lymphoma (Ishida et al., 2006), and breast cancer (Menetrier-Caux et al., 2009). Blockade of CCL22 *in vivo* significantly reduced human TReg cell trafficking to ovarian carcinomas (Curiel et al., 2004). In a study on gastric cancer, CCL22 and CCL17 appeared to be both important in recruiting TReg cells to such tumors as demonstrated by *in vitro* migration assays (Mizukami et al., 2008). Additional observations in this same study further indicated that the levels of intratumoral CCL22 and CCL17 appeared to correlate with increased levels of TReg cell localization within these tumors at early stages of development. Besides the CCR4 chemokine receptor/ligand interaction, CCR5/CCL5 may also selectively recruit TReg cells to pancreatic tumors as shown in both human and murine systems (Tan et al., 2009). In addition, the chemokine CCL20 shows high affinity to TReg cells expressing CCR6 and has been shown to mediate selective CCR6^+^ TReg cell trafficking (Kleinewietfeld et al., 2005). In any instance, both naturally occurring (nTReg) or inducible (iTReg) CD4^+^ TReg cell subpopulations may become enriched within tumors, through a variety of different chemokine receptor/ligand interactions. Furthermore, cytokines and chemokines produced, in part, by either tumor cells, tumor infiltrating lymphocytes, and/or APCs within the tumor milieu may preferentially support such TReg cell expansion, retention, survival, and in some cases, their “further” differentiation and/or change in phenotype (Campbell and Koch, 2011). Following recruitment to sites of inflammation, one of the major functions of CD4^+^ TReg cell subsets is to maintain and/or restore local immune cell homeostasis during polarized Th1, Th2, and Th17 cell-mediated immune responses. This led to the identification and observation that human Th cell subsets and TReg cells appear to undergo functional specialization in parallel resulting in the development of functionally distinct TReg cell subsets capable of co-localizing with and effectively regulating different types of Th cell responses *in vivo* (Hall et al., 2011; Duhen et al., 2012). Although the precise mechanisms by which these various TReg cell subpopulations maintain or restore immune homeostasis at sites of inflammation and/or tumor growth is unknown, it is conceivable that such interactions that involve the local modulation of the naïve, effector, and memory Th cell pools can further influence antitumor responses that may favor either disease progression or regression. In addition, evidence is accumulating in several pre-clinical murine experimental models that a portion of CD4^+^ TReg cells can down-regulate FoxP3 expression and their associated regulatory properties and in some cases acquire an effector cell phenotype that expresses IFN-γ and/or IL-17 (Gavin et al., 2007; Strauss et al., 2007; Miyara et al., 2009; Oldenhove et al., 2009; Martin et al., 2010; Whiteside, 2010; Miyao et al., 2012). Although this concept and process of cellular conversion and/or plasticity among CD4^+^FoxP3^+^ TReg cells remains controversial (Rubtsov et al., 2010), it is clear that the biological properties of CD4 TReg cells and their subpopulations are heterogeneous and influenced by the tumor environment in which they infiltrate (Hamann, 2012; Sainz-Perez et al., 2012).

Alternatively, CD4^+^ TReg cells may possess other underappreciated anti-cancer functions. For example, such cells may have the ability to limit the extent and potential of inflammatory responses to induce tumor development and carcinogenesis. Using a murine herpes viral model, investigators observed that TReg cell-mediated down-modulation of inflammatory responses in secondary lymphoid tissues can actually optimize ensuing immune responses to local infection by more effectively redirecting it to sites of initial infection (Lund et al., 2008). These investigators further suggested that this down-modulation in inflammation within distal secondary lymphoid tissues following local viral infection can facilitate efficient effector T cell migration to sites of primary infection and more effectively promote disease eradication. Conceivably, this can be a concept that can also be applied to local sites of carcinogenesis and primary tumor growth. Moreover, others have suggested that, in colorectal and gastric cancers, CD4^+^ TReg cells may inhibit tumor-promoting inflammatory responses induced by local microbes, which may help explain their presence and favorable association with outcomes to these cancers (Haas et al., 2009; Zamarron and Chen, 2011). Thus the initial views on the role of TReg cells in carcinogenesis, tumor pathology, and tumor immunity appear oversimplified and in fact appear highly context-dependent with respect to their interactions with different components of a dynamic tumor environment.

## Summary and Overall Thoughts

The immune system has the capacity to either obstruct tumor development and deter established tumors, or to promote carcinogenesis and tumor progression. Here, we reviewed how CD4 T cells and their various functionally distinct subpopulations contribute to the antitumor immune response and potentially influence this process. Several key principles emerge. Distinct effector and memory CD4 T cell subsets have important roles in the antitumor response. Key among these roles is the provision of orchestrating and/or regulating other immune cells that result in promoting tumor eradication, long-term tumor immunity, and establishing or rebalancing immune cell homeostasis within the tumor environment. Such roles are mediated, in part, by the production of signature cytokines by specific CD4 T cell lineages and through direct cytotoxic effects on tumor cells. The efficacy of such CD4 effector T cell responses may not only depend on memory cell generation, phenotype, and their functional capacity to interact with other immune cell populations, but also in some cases, on their ability to evolve through “cellular plasticity” within the hostile tumor environment. Conceivably, this trait of “cellular Darwinism” by the various CD4 T cell lineages could endow them with considerable flexibility to procure effective tumor immunity. Alternatively, it appears that such CD4 T cell subsets and their signature cytokines can also contribute and facilitate tumor-promoting activities. This may be due, in part, on the abilities of select CD4 T cell lineages and their subpopulations to modulate effector cell population dynamics and thus affect the balance between effective and ineffective antitumor responses. Achieving an “appropriate cellular balance” appears highly context-dependent. Further studies on the biological activities and mechanisms of how the various polarized CD4 effector and/or memory T cell subsets influence and/or facilitate the immune response as a whole in patients with different types of cancers should further enhance the development of more effective cancer treatment strategies.

## Conflict of Interest Statement

The authors declare that the research was conducted in the absence of any commercial or financial relationships that could be construed as a potential conflict of interest.

## References

[B1] Acosta-Rodriguez E. V. Rivino L. Geginat J. Jarrossay D. Gattorno M. Lanzavecchia A. (2007). Surface phenotype and antigen specificity of human interleukin 17-producing T helper memory cells. Nat. Immunol. 8, 639–646 10.1038/ni1467 17486092

[B2] Agnello D. Lankford C. S. Bream J. Morinobu A. Gadina M. O’Shea J. J. (2003). Cytokines and transcription factors that regulate T helper cell differentiation: new players and new insights. J. Clin. Immunol. 23, 147–161 10.1023/A:1023381027062 12797537

[B3] Aguirre-Ghiso J. A. (2007). Models, mechanisms and clinical evidence for cancer dormancy. Nat. Rev. Cancer 7, 834–846 10.1038/nri2210 17957189 PMC2519109

[B4] Ahmed R. Gray D. (1996). Immunological memory and protective immunity: understanding their relation. Science 272, 54–60 10.1126/science.272.5270.1904 8600537

[B5] Alvaro T. Lejeune M. Salvadó M. T. Bosch R. García J. F. Jaén J. (2005). Outcome in Hodgkin’s lymphoma can be predicted from the presence of accompanying cytotoxic and regulatory T cells. Cancer Res. 11, 1467–1473 10.1158/1078-0432.CCR-04-1869 15746048

[B6] Annunziato F. Cosmi L. Liotta F. Maggi E. Romagnani S. (2012). Defining the human T helper 17 cell phenotype. Trends Immunol. 33, 505–512 10.1016/j.it.2012.05.004 22682163

[B7] Annunziato F. Cosmi L. Santarlasci V. Maggi L. Liotta F. Mazzinghi B. (2007). Phenotypic and functional features of human Th17 cells. J. Exp. Med. 204, 1849–1861 10.1084/jem.20070663 17635957 PMC2118657

[B8] Annunziato F. Romagnani S. (2010). The transient nature of the Th17 phenotype. Eur. J. Immunol. 40, 3312–3316 10.1002/eji.201041145 21110314

[B9] Antonysamy M. A. Fanslow W. C. Fu F. Li W. Qian S. Troutt A. B. (1999). Evidence for a role of IL-17 in organ allograft rejection: IL-17 promotes the functional differentiation of dendritic cell progenitors. J. Immunol. 162, 577–584 9886435

[B10] Appay V. Zaunders J. J. Papagno L. Sutton J. Jaramillo A. Waters A. (2002). Characterization of CD4(+) CTLs ex vivo. J. Immunol. 168, 5954–5958 12023402 10.4049/jimmunol.168.11.5954

[B11] Araki K. Turner A. P. Shaffer V. O. Gangappa S. Keller S. A. Bachmann M. F. (2009). mTOR regulates memory CD8 T-cell differentiation. Nature 460, 108–112 10.1038/nature08155 19543266 PMC2710807

[B12] Arenberg D. A. Kunkel S. L. Polverini P. J. Morris B. Burdick M. D. Glass M. C. (1996). Interferon-gamma-inducible protein 10 (IP-10) is an angiostatic factor that inhibits human non-small cell lung cancer (NSCLC) tumorigenesis and spontaneous metastases. J. Exp. Med. 184, 981–992 10.1084/jem.184.3.981 9064358 PMC2192788

[B13] Bardel E. Larousserie F. Charlot-Rabiega P. Coulomb-L’Herminé A. Devergne O. (2008). Human CD4+CD25+Foxp3+ regulatory T cells do not constitutively express IL-35. J. Immunol. 181, 6898–6905 18981109 10.4049/jimmunol.181.10.6898

[B14] Bates G. J. Fox S. B. Han C. Leek R. D. Garcia J. F. Harris A. L. (2006). Quantification of regulatory T cells enables the identification of high-risk breast cancer patients and those at risk of late relapse. J. Clin. Oncol. 24, 5373–5380 10.1200/JCO.2006.05.9584 17135638

[B15] Beatty G. Paterson Y. (2001). IFN-gamma-dependent inhibition of tumor angiogenesis by tumor-infiltrating CD4 T cells requires tumor responsiveness to IFN-gamma. J. Immunol. 166, 2276–2282 11160282 10.4049/jimmunol.166.4.2276

[B16] Bending D. De la Peña H. Veldhoen M. Phillips J. M. Uyttenhove C. Stockinger B. (2009). Highly purified Th17 cells from BDC2.5NOD mice convert into Th1-like cells in NOD/SCID recipient mice. J. Clin. Invest. 119, 565–572 10.1172/JCI37865 19188681 PMC2648686

[B17] Beriou G. Bradshaw E. M. Lozano E. Costantino C. M. Hastings W. D. Orban T. (2010). TGF-beta induces IL-9 production from human Th17 cells. J. Immunol. 185, 46–54 10.4049/jimmunol.1000356 20498357 PMC2936106

[B18] Berman R. M. Suzuki T. Tahara H. Robbins P. D. Narula S. K. Lotze M. T. (1996). Systemic administration of cellular IL-10 induces an effective, specific, and long-lived immune response against established tumors in mice. J. Immunol. 157, 231–238 8683120

[B19] Berner V. Liu H. Zhou Q. Alderson K. L. Sun K. Weiss J. M. (2007). IFN-gamma mediates CD4? T-cell loss and impairs secondary antitumor responses after successful initial immunotherapy. Nat. Med. 13, 354–360 10.1038/nm1554 17334371

[B20] Bindea G. Mlecnik B. Fridman W. H. Pages F. Galon J. (2010). Natural immunity to cancer in humans. Curr. Opin. Immunol. 22, 215–222 10.1016/j.coi.2010.02.006 20207124

[B21] Blattman J. N. Greenberg P. D. (2004). Cancer immunotherapy: a treatment for the masses. Science 305, 200–205 10.1126/science.1100369 15247469

[B22] Boehm U. Klamp T. Groot M. Howard J. C. (1997). Cellular responses to interferon-gamma. Annu. Rev. Immunol. 15, 749–795 10.1146/annurev.immunol.15.1.749 9143706

[B23] Bollard C. M. Gottschalk S. Leen A. M. Weiss H. Straathof K. C. Carrum G. (2007). Complete responses of relapsed lymphoma following genetic modification of tumor-antigen presenting cells and T-lymphocyte transfer. Blood 110, 2838–2845 10.1182/blood-2007-05-091280 17609424 PMC2018666

[B24] Bonecchi R. Bianchi G. Bordignon P. P. D’Ambrosio D. Lang R. Borsatti A. (1998). Differential expression of chemokine receptors and chemotactic responsiveness of type 1 T helper cells (Th1s) and Th2s. J. Exp. Med. 187, 129–134 10.1084/jem.187.1.129 9419219 PMC2199181

[B25] Boulland M. L. Marquet J. Molinier-Frenkel V. Möller P. Guiter C. Lasoudris F. (2007). Human IL4I1 is a secreted L-phenylalanine oxidase expressed by mature dendritic cells that inhibits T-lymphocyte proliferation. Blood 110, 220–227 10.1182/blood-2006-07-036210 17356132

[B26] Bromberg J. F. Horvath C. M. Wen Z. Schreiber R. D. Darnell J. E. Jr. (1996). Transcriptionally active Stat1 is required for the antiproliferative effects of both interferon alpha and interferon gamma. Proc. Natl. Acad. Sci. U.S.A. 93, 7673–7678 10.1073/pnas.93.15.7673 8755534 PMC38805

[B27] Brown D. M. Kamperschroer C. Dilzer A. M. Roberts D. M. Swain S. L. (2009). IL-2 and antigen dose differentially regulate perforin- and FasL-mediated cytolytic activity in antigen specific CD4+ T cells. Cell. Immunol. 257, 69–79 10.1016/j.cellimm.2009.03.002 19338979 PMC2683476

[B28] Buckner J. H. (2010). Mechanisms of impaired regulation by CD4+CD25+FOXP3+ regulatory T cells in human autoimmune disease. Nat. Rev. Immunol. 10, 849–859 10.1038/nri2889 21107346 PMC3046807

[B29] Campbell D. J. Koch M. A. (2011). Phenotypical and functional specialization of FOXP3+ regulatory T cells. Nat. Rev. Immunol. 11, 119–130 10.1038/nri2916 21267013 PMC3289970

[B30] Cao X. Leonard K. Collins L. I. Cai S. F. Mayer J. C. Payton J. E. (2009). Interleukin 12 stimulates IFN-γ-mediated inhibition of tumor-induced regulatory T-cell proliferation and enhances tumor clearance. Cancer Res. 69, 8700–8709 10.1158/0008-5472.CAN-09-2693 19843867 PMC2783758

[B31] Cardone J. Le Friec G. Vantourout P. Roberts A. Fuchs A. Jackson I. (2010). Complement regulator CD46 temporally regulates cytokine production by conventional and unconventional T cells. Nat. Immunol. 11, 862–871 10.1038/ni.1917 20694009 PMC4011020

[B32] Caretto D. Katzman S. D. Villarino A. V. Gallo E. Abbas A. K. (2010). Cutting edge: the Th1 response inhibits the generation of peripheral regulatory T cells. J. Immunol. 184, 30–34 10.4049/jimmunol.0903412 19949064 PMC2908389

[B33] Carlin J. M. Borden E. C. Sondel P. M. Byrne G. I. (1987). Biologic response-modifier-induced indoleamine 2, 3-dioxygenase activity in human peripheral blood mononuclear cell cultures. J. Immunol. 139, 2414–2418 2443564

[B34] Casazza J. P. Betts M. R. Price D. A. Precopio M. L. Ruff L. E. Brenchley J. M. (2006). Acquisition of direct antiviral effector functions by CMV-specific CD4+ T lymphocytes with cellular maturation. J. Exp. Med. 203, 2865–2877 10.1084/jem.20052246 17158960 PMC2118179

[B35] Chang H. C. Han L. Jabeen R. Carotta S. Nutt S. L. Kaplan M. H. (2009). PU.1 regulates TCR expression by modulating GATA-3 activity. J. Immunol. 183, 4887–4894 10.4049/jimmunol.0804063 19801513 PMC2768261

[B36] Chang H. C. Sehra S. Goswami R. Yao W. Yu Q. Stritesky G. L. (2010). The transcription factor PU.1 is required for the development of IL-9-producing T cells and allergic inflammation. Nat. Immunol. 11, 527–534 10.1038/ni.1867 20431622 PMC3136246

[B37] Chang H. C. Zhang S. Thieu V. T. Slee R. B. Bruns H. A. Laribee R. N. (2005). PU.1 expression delineates heterogeneity in primary Th2 cells. Immunity 22, 693–703 10.1016/j.immuni.2005.03.016 15963784

[B38] Charo I. F. Ransohoff R. M. (2006). The many roles of chemokines and chemokine receptors in inflammation. N. Engl. J. Med. 354, 610–621 10.1056/NEJMra052723 16467548

[B39] Chaturvedi V. Collison L. W. Guy C. S. Workman G. J. Vignali D. A. (2011). Cutting edge: human regulatory T cells require IL-35 to mediate suppression and infectious tolerance. J. Immunol. 186, 6661–6666 10.4049/jimmunol.1100315 21576509 PMC3110563

[B40] Chaudhry A. Samstein R. M. Treuting P. Liang Y. Pils M. C. Heinrich J. M. (2011). Interleukin-10 signaling in regulatory T cells is required for suppression of Th17 cell-mediated inflammation. Immunity 34, 566–578 10.1016/j.immuni.2011.03.018 21511185 PMC3088485

[B41] Cheever M. A. Allison J. P. Ferris A. S. Finn O. J. Hastings B. M. Hecht T. T. (2009). The prioritization of cancer antigens: a national cancer institute pilot project for the acceleration of translational research. Clin. Cancer Res. 15, 5323–5337 10.1158/1078-0432.CCR-09-0737 19723653 PMC5779623

[B42] Chen J. G. Xia J. C. Liang X. T. Pan K. Wang W. Lv L. (2011). Intratumoral expression of IL-17 and its prognostic role in gastric adenocarcinoma patients. Int. J. Biol. Sci. 7, 53–60 21234303 10.7150/ijbs.7.53 PMC3020363

[B43] Chen W. F. Zlotnik A. (1991). IL-10: a novel cytotoxic T cell differentiation factor. J. Immunol. 147, 528–534 1906502

[B44] Chin Y. E. Kitagawa M. Kuida K. Flavell R. A. Fu X. Y. (1997). Activation of the STAT signaling pathway can cause expression of caspase 1 and apoptosis. Mol. Cell. Biol. 17, 5328–5337 9271410 10.1128/mcb.17.9.5328 PMC232383

[B45] Chin Y. E. Kitagawa M. Su W. C. You Z. H. Iwamoto Y. Fu X. Y. (1996). Cell growth arrest and induction of cyclin-dependent kinase inhibitor p21 WAF1/CIP1 mediated by STAT1. Science 272, 719–722 10.1126/science.272.5262.719 8614832

[B46] Chtanova T. Tangye S. G. Newton R. Frank N. Hodge M. R. Rolph M. S. (2004). T follicular helper cells express a distinctive transcriptional profile, reflecting their role as non-Th1/Th2 effector cells that provide help for B cells. J. Immunol. 173, 68–78 15210760 10.4049/jimmunol.173.1.68

[B47] Cole K. E. Strick C. A. Paradis T. J. Ogborne K. T. Loetscher M. Gladue R. P. (1998). Interferon-inducible T cell alpha chemoattractant (I-TAC). J. Exp. Med. 187, 2009–2021 10.1084/jem.187.12.2009 9625760 PMC2212354

[B48] Collison L. W. Chaturvedi V. Henderson A. L. Giacomin P. R. Guy C. Bankoti J. (2010). IL-35-mediated induction of a potent regulatory T cell population. Nat. Immunol. 11, 1093–1101 10.1038/ni.1952 20953201 PMC3008395

[B49] Cope A. Le Friec G. Cardone J. Kemper C. (2011). The Th1 life cycle: molecular control of IFN-γ to IL-10 switching. Trends Immunol. 32, 278–286 10.1016/j.it.2011.03.010 21531623

[B50] Corthay A. Skovseth D. K. Lundin K. U. Røsjø E. Omholt H. Hofgaard P. O. (2005). Primary antitumor immune response mediated by CD4+ T cells. Immunity 22, 371–383 10.1016/j.immuni.2005.02.003 15780993

[B51] Cosmi L. De Palma R. Santarlasci V. Maggi L. Capone M. Frosali F. (2008). Human interleukin 17-producing cells originate from a CD161+CD4+ T cell precursor. J. Exp. Med. 205, 1903–1916 10.1084/jem.20080397 18663128 PMC2525581

[B52] Coughlin C. M. Salhany K. E. Gee M. S. LaTemple D. C. Kotenko S. Ma X. (1998). Tumor cell responses to IFN-gamma affect tumorigenicity and response to IL-12 therapy and antiangiogenesis. Immunity 9, 25–34 10.1016/S1074-7613(00)80585-3 9697833

[B53] Cox M. A. Harrington L. E. Zajac A. J. (2011). Cytokines and the inception of CD8 T cell responses. Trends Immunol. 32, 180–186 10.1016/j.it.2011.01.004 21371940 PMC3074938

[B54] Creagan E. T. Ahmann D. L. Long H. J. Frytak S. Sherwin S. A. Chang M. N. (1987). Phase II study of recombinant interferon-gamma in patients with disseminated malignant melanoma. Cancer Treat. Rep. 71, 843–844 3113730

[B55] Cruz-Guilloty F. Pipkin M. E. Djuretic I. M. Levanon D. Lotem J. Lichtenheld M. G. (2009). Runx3 and T-box proteins cooperate to establish the transcriptional program of effector CTLs. J. Exp. Med. 206, 51–59 10.1084/jem.20081242 19139168 PMC2626671

[B56] Curiel T. J. Coukos G. Zou L. Alvarez X. Cheng P. Mottram P. (2004). Specific recruitment of regulatory T cells in ovarian carcinoma fosters immune privilege and predicts reduced survival. Nat. Med. 10, 942–949 10.1038/nm1093 15322536

[B57] Curiel T. J. Wei S. Dong H. Alvarez X. Cheng P. Mottram P. (2003). Blockade of B7-H1 improves myeloid dendritic cell-mediated antitumor immunity. Nat. Med. 9, 562–567 10.1038/nm863 12704383

[B58] Dash R. Richards J. E. Su Z. Z. Bhutia S. K. Azab B. Rahmani M. (2010). Mechanism by which Mcl-1 regulates cancer-specific apoptosis triggered by mda-7/IL-24, an IL-10-related cytokine. Cancer Res. 70, 5034–5045 10.1158/1538-7445.AM10-5034 20501829 PMC3171699

[B59] de Waal Malefyt R. Yssel H. de Vries J. E. (1993). Direct effects of IL-10 on subsets of human CD4þ T cell clones and resting T cells. Specific inhibition of IL-2 production and proliferation. J. Immunol. 150, 4754–4765 7684412

[B60] deLeeuw R. J. Kost S. E. Kakal J. A. Nelson B. H. (2012). The prognostic value of FoxP3+ tumor-infiltrating lymphocytes in cancer: a critical review of the literature. Clin. Cancer Res. 18, 3022–3029 10.1158/1078-0432.CCR-11-3216 22510350

[B61] Derhovanessian E. Adams V. Hähnel K. Groeger A. Pandha H. Ward S. (2009). Pretreatment frequency of circulating IL-17+ CD4+ T-cells, but not Tregs, correlates with clinical response to whole-cell vaccination in prostate cancer patients. Int. J. Cancer 125, 1372–1379 10.1002/ijc.24497 19533748

[B62] Dobrzanski M. J. Rewers-Felkins K. A. Samad K. A. Quinlin I. S. Phillips C. A. Robinson W. (2009). Autologous MUC1-specific Th1 effector cell immunotherapy induces differential levels of systemic TReg cell populations that result in increased ovarian cancer patient survival. Clin. Immunol. 133, 333–352 10.1016/j.clim.2009.08.007 19762283 PMC2805085

[B63] Dobrzanski M. J. Rewers-Felkins K. A. Samad K. A. Quinlin I. S. Phillips C. A. Robinson W. (2011). Immunotherapy with IL-10- and IFN-γ-producing CD4 effector cells modulate “Natural” and “Inducible” CD4 TReg cell subpopulation levels: observations in four cases of patients with ovarian cancer. Cancer Immunol. Immunother. 61, 839–854 10.1007/s00262-011-1128-x 22083345 PMC3315597

[B64] Dong H. Strome S. E. Salomao D. R. Tamura H. Hirano F. Flies D. B. (2002). Tumor-associated B7-H1 promotes T-cell apoptosis: a potential mechanism of immune evasion. Nat. Med. 8, 793–800 12091876 10.1038/nm730

[B65] Dougan M. Dranoff G. (2009). Immune therapy for cancer. Annu. Rev. Immunol. 27, 83–117 10.1146/annurev.immunol.021908.132544 19007331

[B66] Dudley M. E. Wunderlich J. R. Robbins P. F. Yang J. C. Hwu P. Schwartzentruber D. J. (2002). Cancer regression and autoimmunity in patients after clonal repopulation with antitumor lymphocytes. Science 298, 850–854 10.1126/science.1076514 12242449 PMC1764179

[B67] Dudley M. E. Yang J. C. Sherry R. Hughes M. S. Royal R. Kammula U. (2008). Adoptive cell therapy for patients with metastatic melanoma: evaluation of intensive myeloablative chemoradiation preparative regimens. J. Clin. Oncol. 26, 5233–5239 10.1200/JCO.2008.16.5449 18809613 PMC2652090

[B68] Duhen T. Duhen R. Lanzavecchia A. Sallusto F. Campbell D. J. (2012). Functionally distinct subsets of human FOXP3+ Treg cells that phenotypically mirror effector Th cells. Blood 119, 4430–4440 10.1182/blood-2011-11-392324 22438251 PMC3362361

[B69] Duhen T. Geiger R. Jarrossay D. Lanzavecchia A. Sallusto F. (2009). Production of interleukin 22 but not interleukin 17 by a subset of human skin-homing memory T cells. Nat. Immunol. 10, 857–863 10.1038/ni.1767 19578369

[B70] Dunn G. P. Koebel C. M. Schreiber R. D. (2006). Interferons, immunity and cancer immunoediting. Nat. Rev. Immunol. 6, 836–848 10.1038/nri1961 17063185

[B71] Ealick S. E. Cook W. J. Vijay-Kumar S. Carson M. Nagabhushan T. L. Trotta P. P. (1991). Three-dimensional structure of recombinant human interferon-gamma. Science 252, 698–702 10.1126/science.1902591 1902591

[B72] Emdad L. Lebedeva I. V. Su Z. Z. Gupta P. Sauane M. Dash R. (2009). Historical perspective and recent insights into our understanding of the molecular and biochemical basis of the antitumor properties of mda-7/IL-24. Cancer Biol. Ther. 8, 391–400 10.4161/cbt.8.4.8156 19276652

[B73] Emmerich J. Mumm J. B. Chan I. H. LaFace D. Truong H. McClanahan T. (2012). IL-10 directly activates and expands tumor-resident CD8(+) T cells without de novo infiltration from secondary lymphoid organs. Cancer Res. 72, 3570–3581 10.1158/0008-5472.CAN-12-0721 22581824

[B74] Endo Y. Iwamura C. Kuwahara M. Suzuki A. Sugaya K. Tumes D. J. (2011). Eomesodermin controls interleukin-5 production in memory T helper 2 cells through inhibition of activity of the transcription factor GATA3. Immunity 35, 733–745 10.1016/j.immuni.2011.08.017 22118525

[B75] Enk A. H. Jonuleit H. Saloga J. Knop J. (1997). Dendritic cells as mediators of tumor-induced tolerance in metastatic melanoma. Int. J. Cancer 73, 309–316 10.1002/(SICI)1097-0215(19971104)73:3<309::AID-IJC1>3.3.CO;2-B 9359474

[B76] Erdman S. E. Sohn J. J. Roa V. P. Nambiar P. R. Ge Z. Fox J. G. (2005). CD4+CD25+ regulatory lymphocytes induce regression of intestinal tumors in Apc^Min/+^ mice. Cancer Res. 65, 3998–4004 10.1158/0008-5472.CAN-04-3104 15899788

[B77] Ernstoff M. S. Trautman T. Davis C. A. Reich S. D. Witman P. Balser J. (1987). A randomized phase I/II study of continuous versus intermittent intravenous interferon gamma in patients with metastatic melanoma. J. Clin. Oncol. 5, 1804–1810 3119786 10.1200/JCO.1987.5.11.1804

[B78] Eyerich S. Eyerich K. Pennino D. Carbone T. Nasorri F. Pallotta S. (2009). Th22 represent a distinct subset of human T cell subset involved in epidermal immunity and remodeling. J. Clin. Invest. 119, 3573–3585 19920355 10.1172/JCI40202 PMC2786807

[B79] Fallarino F. Grohmann U. Hwang K. W. Orabona C. Vacca C. Bianchi R. (2003). Modulation of tryptophan catabolism by regulatory T cells. Nat. Immunol. 4, 1206–1212 10.1038/ni1003 14578884

[B80] Farber J. M. (1990). A macrophage mRNA selectively induced by gamma-interferon encodes a member of the platelet factor 4 family of cytokines. Proc. Natl. Acad. Sci. U.S.A. 87, 5238–5242 10.1073/pnas.87.14.5238 2115167 PMC54298

[B81] Feighery C. Stastny P. (1979). HLA-D region-associated determinants serve as targets for human cell mediated lysis. J. Exp. Med. 149, 485–494 10.1084/jem.149.2.485 84045 PMC2184802

[B82] Fisher P. B. (2005). Is mda-7/IL-24 a “magic bullet” for cancer? Cancer Res. 65, 10128–10138 10.1158/0008-5472.CAN-05-3127 16287994

[B83] Frederiksen K. S. Lundsgaard D. Freeman J. A. Hughes S. D. Holm T. L. Skrumsager B. K. (2008). IL-21 induces in vivo immune activation of NK cells and CD8 T cells in patients with metastatic melanoma and renal cell carcinoma. Cancer Immunol. Immunother. 57, 1439–1449 10.1007/s00262-008-0479-4 18286285 PMC2491425

[B84] Fridman W. H. Mlecnik B. Bindea1 G. Pages F. Galon J. (2011). Immunosurveillance in human non-viral cancers. Curr. Opin. Immunol. 23, 272–278 10.1016/j.coi.2010.12.011 21237631

[B85] Fujii S. Shimizu K. Shimizu T. Lotze M. T. (2001). Interleukin-10 promotes the maintenance of antitumor CD8 T-cell effector function in situ. Blood 98, 2143–2151 10.1182/blood.V98.7.2143 11568001

[B86] Gabrilovich D. I. Ostrand-Rosenberg S. Bronte V. (2012). Coordinated regulation of myeloid cells by tumours. Nat. Rev. Immunol. 12, 253–268 10.1038/nri3175 22437938 PMC3587148

[B87] Gaffen S. L. (2011). Recent advances in the IL-17 cytokine family. Curr. Opin. Immunol. 23, 613–619 10.1016/j.coi.2011.07.006 21852080 PMC3190066

[B88] Galon J. Costes A. Sanchez-Cabo F. Kirilovsky A. Mlecnik B. Lagorce-Pages C. (2006). Type, density, and location of immune cells within human colorectal tumors predict clinical outcome. Science 313, 1960–1964 10.1126/science.1129139 17008531

[B89] Gavin M. A. Rasmussen J. P. Fontenot J. D. Vasta V. Manganiello V. C. Beavo J. A. (2007). Foxp3-dependent programme of regulatory T-cell differentiation. Nature 445, 771–775 10.1038/nature05543 17220874

[B90] Giovarelli M. Musiani P. Modesti A. Dellabona P. Casorati G. Allione A. (1995). Local release of IL-10 by transfected mouse mammary adenocarcinoma cells does not suppress but enhances antitumor reaction and elicits a strong cytotoxic lymphocyte and antibody-dependent immune memory. J. Immunol. 155, 3112–3123 7673726

[B91] Grazia-Roncarolo M. Gregori S. Battaglia M. Bacchetta R. Fleischhauer K. Levings M. K. (2006). IL-10-secreting type 1 regulatory T cells in rodents and humans. Immunol. Rev. 212, 28–50 10.1111/j.0105-2896.2006.00420.x 16903904

[B92] Green D. R. Ferguson T. A. (2001). The role of Fas ligand in immune privilege. Nat. Rev. Mol. Cell Biol. 10.1038/35103104 11733771

[B93] Grivennikov S. Greten F. R. Karin M. (2010). Immunity, inflammation, and cancer. Cell 140, 883–899 10.1016/j.cell.2010.01.025 20303878 PMC2866629

[B94] Groux H. Bigler M. de Vries J. E. Roncarolo M. G. (1998). Inhibitory and stimulatory effects of IL-10 on human CD8 T cells. J. Immunol. 160, 3188–3193 9531274

[B95] Groux H. O’Garra A. Bigler M. Rouleau M. Antonenko S. de Vries J. E. (1997). A CD4^+^ T cell subset inhibits antigen-specific T cell responses and prevents colitis. Nature 389, 737–742 10.1038/39614 9338786

[B96] Haas M. Dimmler A. Hohenberger W. Grabenbauer G. G. Niedobitek G. Distel L. V. (2009). Stromal regulatory T-cells are associated with a favourable prognosis in gastric cancer of the cardia. BMC Gastroenterol. 9, 65–71 10.1186/1471-230X-9-65 19732435 PMC2749861

[B97] Haas J. Schopp L. Storch-Hagenlocher B. Fritzsching B. Jacobi C. Milkova L. (2008). Specific recruitment of regulatory T cells into the CSF in lymphomatous and carcinomatous meningitis. Blood 111, 761–766 10.1182/blood-2007-08-104877 17967942

[B98] Hall B. M. Verma N. D. Tran G. T. Hodgkinson S. J. (2011). Distinct regulatory CD4+T cell subsets; differences between naïve and antigen specific T regulatory cells. Curr. Opin. Immunol. 23, 641–647 10.1016/j.coi.2011.07.012 21840184

[B99] Hamaï A. Pignon P. Raimbaud I. Duperrier-Amouriaux K. Senellart H. Hiret S. (2012). Human T_H_17 immune cells specific for the tumor antigen MAGE-A3 convert to IFN-γ-secreting cells as they differentiate into effector T cells in vivo. Cancer Res. 72, 1059–1063 10.1158/0008-5472.CAN-11-3432 22253231

[B100] Hamann A. (2012). Regulatory T cells stay the course. Immunity 36, 161–163 10.1016/j.immuni.2012.02.004 22365661

[B101] Haynes N. M. Allen C. D. Lesley R. Ansel K. M. Killeen N. Cyster J. G. (2007). Role of CXCR5 and CCR7 in follicular Th cell positioning and appearance of a programmed cell death gene-1high germinal center-associated subpopulation. J. Immunol. 179, 5099–5108 17911595 10.4049/jimmunol.179.8.5099

[B102] Hirai H. Tanaka K. Yoshie O. Ogawa K. Kenmotsu K. Takamori Y. (2001). Prostaglandin D2 selectively induces chemotaxis in T helper type 2 cells, eosinophils, and basophils via seven-transmembrane receptor. J. Exp. Med. 193, 255–262 10.1084/jem.193.2.255 11208866 PMC2193345

[B103] Hirota K. Duarte J. H. Veldhoen M. Hornsby E. Li Y. Cua D. J. (2011). Fate mapping of IL-17-producing T cells in inflammatory responses. Nat. Immunol. 12, 255–263 10.1038/ni.1993 21278737 PMC3040235

[B104] Hirschhorn-Cymerman D. Budhu S. Kitano S. Liu C. Zhao F. Zhong H. (2012). Induction of tumoricidal function in CD4^+^ T cells is associated with concomitant memory and terminally differentiated phenotype. J. Exp. Med. 209, 2113–2126 10.1084/jem.20120532 23008334 PMC3478933

[B105] Ho W. Y. Yee C. Greenberg P. D. (2002). Adoptive therapy with CD8+ T cells: it may get by with a little help from its friends. J. Clin. Invest. 110, 1415–1417 10.1172/JCI17214 12438439 PMC151827

[B106] Hobeika A. C. Etienne W. Torres B. A. Johnson H. M. Subramaniam P. S. (1999). IFN-gamma induction of p21(WAF1) is required for cell cycle inhibition and suppression of apoptosis. J. Interferon Cytokine Res. 19, 1351–1361 10.1089/107999099312812 10638704

[B107] Honorati M. C. Neri S. Cattini L. Facchini A. (2006). Interleukin-17, a regulator of angiogenic factor release by synovial fibroblasts. Osteoarthritis Cartilage 14, 345–352 10.1016/j.joca.2005.10.004 16311048

[B108] Houssiau F. A. Schandene L. Stevens M. Cambiaso C. Goldman M. van Snick J. (1995). A cascade of cytokines is responsible for IL-9 expression in human T cells. J. Immunol. 154, 2624–2630 7876537

[B109] Huber S. Gagliani N. Esplugues E. O’Connor W. Huber F. J. Chaudhry A. (2011). Th17 cells express interleukin-10 receptor and are controlled by Foxp3 and Foxp3+ regulatory CD4+ T cells in an interleukin-10-dependent manner. Immunity 34, 554–565 10.1016/j.immuni.2011.01.020 21511184 PMC3113617

[B110] Hunder N. N. Wallen H. Cao J. Hendricks D. W. Reilly J. Z. Rodmyre R. (2008). Treatment of metastatic melanoma with autologous CD4+ T cells against NY-ESO-1. N. Engl. J. Med. 358, 2698–2703 10.1056/NEJMoa0800251 18565862 PMC3277288

[B111] Hung K. Hayashi R. Lafond-Walker A. Lowenstein C. Pardoll D. Levitsky H. (1998). The central role of CD4+ T cells in the antitumor immune response. J. Exp. Med. 188, 2357–2368 10.1084/jem.188.12.2357 9858522 PMC2212434

[B112] Hwang E. S. Szabo S. J. Schwartzberg P. L. Glimcher L. H. (2005). T helper cell fate specified by kinase-mediated interaction of T-bet with GATA-3. Science 307, 430–433 10.1126/science.1103336 15662016

[B113] Iellem A. Mariani M. Lang R. Recalde H. Panina-Bordignon P. Sinigaglia F. (2001). Unique chemotactic response profile and specific expression of chemokine receptors CCR4 and CCR8 by CD4(+) CD25(+) regulatory T cells. J. Exp. Med. 194, 847–853 10.1084/jem.194.6.847 11560999 PMC2195967

[B114] Intlekofer A. M. Banerjee A. Takemoto N. Gordon S. M. DeJong C. S. Shin H. (2008). Anomalous type 17 response to viral infection by CD8+ T cells lacking T-bet and eomesodermin. Science 321, 408–411 10.1126/science.1159806 18635804 PMC2807624

[B115] Intlekofer A. M. Takemoto N. Wherry E. J. Longworth S. A. Northrup J. T. Palanivel V. R. (2005). Effector and memory CD8+ T cell fate coupled by T-bet and eomesodermin. Nat. Immunol. 6, 1236–1244 10.1038/ni1268 16273099

[B116] Ishida T. Ishii T. Inagaki A. Yano H. Komatsu H. Iida S. (2006). Specific recruitment of CC chemokine receptor 4-positive regulatory T cells in Hodgkin lymphoma fosters immune privilege. Cancer Res. 66, 5716–5722 10.1158/0008-5472.CAN-06-0261 16740709

[B117] Jabeen R. Kaplan M. H. (2012). The symphony of the ninth: the development and function of Th9 cells. Curr. Opin. Immunol. 24, 303–307 10.1016/j.coi.2012.02.001 22365614 PMC3368035

[B118] Jankovic D. Kugler D. G. Sher A. (2010). IL-10 production by CD4+ effector T cells: a mechanism for self-regulation. Mucosal Immunol. 3, 239–246 10.1038/mi.2010.8 20200511 PMC4105209

[B119] Janssen E. M. Lemmens E. E. Wolfe T. Christen U. von Herrath M. G. Schoenberger S. P. (2003). CD4+ T cells are required for secondary expansion and memory in CD8+ T lymphocytes. Nature 421, 852–856 10.1038/nature01441 12594515

[B120] Jenner R. G. Townsend M. J. Jackson I. Sun K. Bouwman R. D. Young R. A. (2009). The transcription factors T-bet and GATA-3 control alternative pathways of T-cell differentiation through a shared set of target genes. Proc. Natl. Acad. Sci. U.S.A. 106, 17876–17881 10.1073/pnas.0909357106 19805038 PMC2764903

[B121] Jiang R. Tan Z. Deng L. Chen Y. Xia Y. Gao Y. (2011). Interleukin-22 promotes human hepatocellular carcinoma by activation of STAT3. Hepatology 54, 900–909 10.1002/hep.24486 21674558

[B122] Jinquan T. Larsen C. G. Gesser B. Matsushima K. Thestrup-Pedersen K. (1993). Human IL-10 is a chemoattractant for CD8 T lymphocytes and an inhibitor of IL-8-induced CD4 T lymphocyte migration. J. Immunol. 151, 4545–4551 8104996

[B123] Joshi N. S. Cui W. Chandele A. Lee H. K. Urso D. R. Hagman J. (2005). Inflammation directs memory precursor and short-lived effector CD8+ T cell fates via the graded expression of T-bet transcription factor. Immunity 27, 281–295 10.1016/j.immuni.2007.07.010 17723218 PMC2034442

[B124] Joss A. Akdis M. Faith A. Blaser K. Akdis C. A. (2000). IL-10 directly acts on T cells by specifically altering the CD28 co-stimulation pathway. Eur. J. Immunol. 30, 1683–1690 10.1002/1521-4141(200006)30:6<1683::AID-IMMU1683>3.0.CO;2-A 10898505

[B125] Jovanovic D. V. Di Battista J. A. Martel-Pelletier J. Jolicoeur F. C. He Y. Zhang M. (1998). IL-17 stimulates the production and expression of proinflammatory cytokines, IL- and TNF-, by human macrophages. J. Immunol. 160, 3513–3521 9531313

[B126] Kaplan M. H. Schindler U. Smiley S. T. Grusby M. J. (1996). Stat6 is required for mediating responses to IL-4 and for development of Th2 cells. Immunity 4, 313–319 10.1016/S1074-7613(00)80439-2 8624821

[B127] Kawakami Y. Tomimori Y. Yumoto K. Hasegawa S. Ando T. Tagaya Y. (2009). Inhibition of NK cell activity by IL-17 allows vaccinia virus to induce severe skin. J. Exp. Med. 206, 1219–1225 10.1084/jem.20082835 19468065 PMC2715052

[B128] Kennedy R. Celis E. (2008). Multiple roles for CD4 T cells in the antitumor immune response. Immunol. Rev. 222, 129–144 10.1111/j.1600-065X.2008.00616.x 18363998

[B129] Kershaw M. H. Westwood J. A. Parker L. L. Wang G. Eshhar Z. Mavroukakis S. A. (2006). A phase I study on adoptive immunotherapy using gene-modified T cells for ovarian cancer. Clin. Cancer Res. 12, 6106–6115 10.1158/1078-0432.CCR-06-1183 17062687 PMC2154351

[B130] Kesselring R. Thiel A. Pries R. Trenkle T. Wollenberg B. (2010). Human Th17 cells can be induced through head and neck cancer and have a functional impact on HNSCC development. Br. J. Cancer 103, 1245–1254 10.1038/sj.bjc.6605891 20877351 PMC2967064

[B131] Kim C. H. Rott L. Kunkel E. J. Genovese M. C. Andrew D. P. Wu L. (2001). Rules of chemokine receptor association with T cell polarization in vivo. J. Clin. Invest. 108, 1331–1339 10.1172/JCI200113543 11696578 PMC209443

[B132] Kim J. Modlin R. L. Moy R. L. Dubinett S. McHugh T. Nickoloff B. J. (1995). IL-10 production in cutaneous basal and squamous cell carcinomas: a mechanism for evading the local T cell immune response. J. Immunol. 155, 2240–2247 7636270

[B133] Kleinewietfeld M. Puentes F. Borsellino G. Battistini L. Rötzschke O. Falk K. (2005). CCR6 expression defines regulatory effector/memory-like cells within the CD25+CD4+ T-cell subset. Blood 105, 2877–2886 10.1182/blood-2004-07-2505 15613550

[B134] Kleinschek M. A. Boniface K. Sadekova S. Grein J. Murphy E. E. Turner S. P. (2009). Circulating and gut-resident human Th17 cells express CD161 and promote intestinal inflammation. J. Exp. Med. 206, 525–534 10.1084/jem.20081712 19273624 PMC2699125

[B135] Kloke O. Wandl U. Opalka B. Moritz T. Nagel-Hiemke M. Franz T. (1992). A prospective randomized comparison of single-agent interferon (IFN)-alpha with the combination of IFN-alpha and low-dose IFN-gamma in chronic myelogenous leukaemia. Eur. J. Haematol. 48, 93–98 1547881 10.1111/j.1600-0609.1992.tb00572.x

[B136] Kondo A. Yamashita T. Tamura H. Zhao W. Tsuji T. Shimizu M. (2010). Interferon-gamma and tumor necrosis factor-alpha induce an immunoinhibitory molecule, B7-H1, via nuclear factor-kappaB activation in blasts in myelodysplastic syndromes. Blood 116, 1124–1131 10.1182/blood-2009-12-255125 20472834 PMC3375140

[B137] Kopp W. C. Smith J. W. Ewel C. H. Alvord W. G. Main C. Guyre P. M. (1993). Immunomodulatory effects of interferon-gamma in patients with metastatic malignant melanoma. J. Immunother. Emphasis Tumor Immunol. 13, 181–190 10.1097/00002371-199304000-00005 8471592

[B138] Kowalzick L. Weyer U. Lange P. Breitbart E. W. (1990). Systemic therapy of advanced metastatic malignant melanoma with a combination of fibroblast interferon-beta and recombinant interferon-gamma. Dermatologica 181, 298–303 10.1159/000247830 2127402

[B139] Kryczek I. Banerjee M. Cheng P. Vatan L. Szeliga W. Wei S. (2009a). Phenotype, distribution, generation, and functional and clinical relevance of Th17 cells in the human tumour environments. Blood 114, 1141–1149 10.1182/blood-2009-03-208249 19470694 PMC2723011

[B140] Kryczek I. Wei S. Szeliga W. Vatan L. Zou W. (2009b). Endogenous IL-17 contributes to reduced tumor growth and metastasis. Blood 114, 357–359 10.1182/blood-2009-03-208249 19289853 PMC2714210

[B141] Kryczek I. Wei S. Zou L. Altuwaijri S. Szeliga W. Kolls J. (2007). Cutting edge: Th17 and regulatory T cell dynamics and the regulation by IL-2 in the tumor microenvironment. J. Immunol. 178, 6730–6733 17513719 10.4049/jimmunol.178.11.6730

[B142] Kryczek I. Zou L. Rodriguez P. Zhu G. Wei S. Mottram P. (2006). B7-H4 expression identifies a novel suppressive macrophage population in human ovarian carcinoma. J. Exp. Med. 203, 871–881 10.1084/jem.20050930 16606666 PMC2118300

[B143] Kurata H. Lee H. J. O’Garra A. Arai N. (1999). Ectopic expression of activated Stat6 induces the expression of Th2-specific cytokines and transcription factors in developing Th1 cells. Immunity 11, 677–688 10.1016/S1074-7613(00)80142-9 10626890

[B144] Kurte M. Lopez M. Aguirre A. Escobar A. Aguillon J. C. Charo J. (2004). A synthetic peptide homologous to functional domain of human IL-10 down-regulates expression of MHC class I and transporter associated with antigen processing 1/2 in human melanoma cells. J. Immunol. 173, 1731–1737 15265902 10.4049/jimmunol.173.3.1731

[B145] Kurzrock R. Talpaz M. Kantarjian H. Walters R. Saks S. Trujillo J. M. (1987). Therapy of chronic myelogenous leukemia with recombinant interferon-gamma. Blood 70, 943–947 3115339

[B146] Lauw F. N. Pajkrt D. Hack C. E. Kurimoto M. van Deventer S. J. van der Poll T. (2000). Proinflammatory effects of IL-10 during human endotoxemia. J. Immunol. 165, 2783–2789 10946310 10.4049/jimmunol.165.5.2783

[B147] Lazarevic V. Chen X. Shim J. H. Hwang E. S. Jang E. Bolm A. N. (2011). T-bet represses TH17 differentiation by preventing Runx1-mediated activation of the gene encoding RORgt. Nat. Immunol. 12, 96–104 10.1038/ni.1969 21151104 PMC3077962

[B148] Lazarevic V. Glimcher L. H. (2011). T-bet in disease. Nat. Immunol. 12, 597–606 10.1038/ni.1969 21685955 PMC6290474

[B149] Le D. T. Jaffee E. M. (2012). Regulatory T-cell modulation using cyclophosphamide in vaccine approaches: a current perspective. Cancer Res. 72, 3439–3444 10.1158/0008-5472.CAN-11-3912 22761338 PMC3399042

[B150] Lebedeva I. V. Emdad L. Su Z. Z. Gupta P. Sauane M. Sarkar D. (2007). mda-7/IL-24, novel anticancer cytokine: focus on bystander antitumor, radiosensitization and antiangiogenic properties and overview of the phase I clinical experience (Review). Int. J. Oncol. 31, 985–1007 17912425

[B151] Lee J. W. Wang P. Kattah M. G. Youssef S. Steinman L. DeFea K. (2008). Differential regulation of chemokines by IL-17 in colonic epithelial cells. J. Immunol. 181, 6536–6545 18941244 10.4049/jimmunol.181.9.6536

[B152] Lee Y. K. Turner H. Maynard C. L. Oliver J. R. Chen D. Elson C. O. (2009). Late developmental plasticity in the T helper 17 lineage. Immunity 30, 92–107 10.1016/j.immuni.2008.11.005 19119024 PMC3607320

[B153] Levine B. L. Bernstein W. B. Aronson N. E. Schlienger K. Cotte J. Perfetto S. (2002). Adoptive transfer of costimulated CD4^+^ T cells induces expansion of peripheral T cells and decreased CCR5 expression in HIV infection. Nat. Med. 8, 47–53 10.1038/nm0102-47 11786906

[B154] Li X. Ye D. Xie X. Chen H. Lu W. (2005). Proportions of CD4+CD25+ regulatory T cells is increased in patients with ovarian cancer. Cancer Invest. 23, 399–403 10.1081/CNV-58815 16193639

[B155] Liu J. Hamrouni A. Wolowiec D. Coiteux V. Kuliczkowski K. Hetuin D. (2007). Plasma cells from multiple myeloma patients express B7-H1 (PD-L1) and increase expression after stimulation with IFN-{gamma} and TLR ligands via a MyD88-, TRAF6-, and MEK-dependent pathway. Blood 110, 296–304 10.1182/blood-2006-11-058594 17363736

[B156] Martin-Orozco N. Chung Y. Chang S. H. Wang Y. H. Dong C. (2009b). Th17 cells promote pancreatic inflammation but only induce diabetes efficiently in lymphopenic hosts after conversion into Th1 cells. Eur. J. Immunol. 39, 216–224 10.1002/eji.200838475 19130584 PMC2755057

[B157] Liu L. Zhang W. Qi X. Li H. Yu J. Wei S. (2012a). Randomized study of autologous cytokine-induced killer cell immunotherapy in metastatic renal carcinoma. Clin. Cancer Res. 18, 1751–1759 10.1158/1078-0432.CCR-11-1973 22275504

[B158] Liu X. Yan X. Zhong B. Nurieva R. I. Wang A. Wang X. (2012b). Bcl6 expression specifies the T follicular helper cell program in vivo. J. Exp. Med. 209, 1841–1852 10.1084/jem.20120219 22987803 PMC3457730

[B159] Liu X. S. Leerberg J. MacDonald K. Leggatt G. R. Frazer I. H. (2009). IFN-γ promotes generation of IL-10 secreting CD4 T cells that suppress generation of CD8 responses in an antigen-experienced host. J. Immunol. 183, 51–58 10.4049/jimmunol.0990046 19535638

[B160] Liyanage U. K. Moore T. T. Joo H. Tanaka Y. Herrmann V. Doherty G. (2002). Prevalence of regulatory T cells is increased in peripheral blood and tumor microenvironment of patients with pancreas or breast adenocarcinoma. J. Immunol. 169, 2756–2761 12193750 10.4049/jimmunol.169.5.2756

[B161] Loddenkemper C. Schernus M. Noutsias M. Stein H. Thiel E. Nagorsen D. (2006). In situ analysis of FoxP3 regulatory T cells in human colorectal cancer. J. Transl. Med. 4, 52–59 10.1186/1479-5876-4-52 17166272 PMC1764431

[B162] Loercher A. E. Nash M. A. Kavanagh J. J. Platsoucas C. D. Freedman R. S. (1999). Identification of an IL-10-producing HLA-DR-negative monocyte subset in the malignant ascites of patients with ovarian carcinoma that inhibits cytokine protein expression and proliferation of autologous T cells. J. Immunol. 163, 6251–6260 10570318

[B163] Loetscher P. Uguccioni M. Bordoli L. Baggiolini M. Moser B. Chizzolini C. (1998). CCR5 is characteristic of Th1 lymphocytes. Nature 391, 344–345 10.1038/34812 9450746

[B164] Lund J. M. Hsing L. Pham T. T. Rudensky A. Y. (2008). Coordination of early protective immunity to viral infection by regulatory T cells. Science 320, 1220–1224 10.1126/science.1155209 18436744 PMC2519146

[B165] Luster A. D. Leder P. (1993). IP-10, a C-X-C-chemokine, elicits a potent thymus-dependent antitumor response in vivo. J. Exp. Med. 178, 1057–1065 10.1084/jem.178.3.1057 8350046 PMC2191174

[B166] Luster A. D. Ravetch J. V. (1987). Biochemical characterization of a gamma interferon-inducible cytokine (IP-10). J. Exp. Med. 1987, 1084–1097 10.1084/jem.166.4.1084 2443596 PMC2188708

[B167] Luther S. A. Cyster J. G. (2001). Chemokines as regulators of T cell differentiation. Nat. Immunol. 2, 102–107 10.1038/84205 11175801

[B168] Ma C. S. Deenick E. K. Batten M. Tangye S. G. (2012). The origins, function, and regulation of T follicular helper cells. J. Exp. Med. 209, 1241–1253 10.1084/jem.20120994 22753927 PMC3405510

[B169] Manel N. Unutmaz D. Littmanet D. R. (2008). The differentiation of human T(H)-17 cells requires transforming growth factor-beta and induction of the nuclear receptor RORγt. Nat. Immunol. 9, 641–649 10.1038/ni.1610 18454151 PMC2597394

[B170] Marshall H. D. Chandele A. Jung Y. W. Meng H. Poholek A. C. Parish I. A. (2011). Differential expression of Ly6C and T-bet distinguish effector and memory Th1 CD4(+) cell properties during viral infection. Immunity 35, 633–646 10.1016/j.immuni.2011.08.016 22018471 PMC3444169

[B171] Marshall N. A. Galvin K. C. Corcoran A. M. Boon L. Higgs R. Mills K. H. (2012). Immunotherapy with PI3K inhibitor and Toll-like receptor agonist induces IFN-γ+IL-17+ polyfunctional T cells that mediate rejection of murine tumors. Cancer Res. 72, 581–591 10.1158/0008-5472.CAN-11-0307 22158905

[B172] Martin F. Ladoire S. Mignot G. Apetoh L. Ghiringhelli F. (2010). Human FOXP3 and cancer. Oncogene 29, 4121–4129 10.1038/onc.2010.174 20498631

[B173] Martin-Orozco N. Muranski P. Chung Y. Yang X. O. Yamazaki T. Lu S. (2009). T helper 17 cells promote cytotoxic T cell activation in tumor immunity. Immunity 31, 787–798 10.1016/j.immuni.2009.09.014 19879162 PMC2787786

[B174] Mattes J. Hulett M. Xie W. Hogan S. Rothenberg M. E. Foster P. (2003). Immunotherapy of cytotoxic T cell-resistant tumors by T helper 2 cells an eotaxin and STAT6-dependent process. J. Exp. Med. 197, 387–393 10.1084/jem.20021683 12566422 PMC2193835

[B175] McKinstry K. K. Strutt T. M. Swain S. L. (2010). The potential of CD4 T-cell memory. Immunology 130, 1–9 10.1111/j.1365-2567.2010.03259.x 20331470 PMC2855787

[B176] Medzhitov R. (2008). Origin and physiological roles of inflammation. Nature 454, 428–435 10.1038/nature07201 18650913

[B177] Mellor A. L. Munn D. H. (2008). Creating immune privilege: active local suppression that benefits friends, but protects foes. Nat. Rev. Immunol. 8, 74–80 10.1038/nri2233 18064049

[B178] Menetrier-Caux C. Gobert M. Caux C. (2009). Differences in tumor regulatory T-cell localization and activation status impact patient outcome. Cancer Res. 69, 7895–7898 10.1158/0008-5472.CAN-09-1642 19808962

[B179] Meng R. D. El-Deiry W. S. (2001). p53-independent upregulation of *KILLER/DR5* TRAIL receptor expression by glucocorticoids and interferon-gamma. Exp. Cell Res. 262, 154–169 10.1006/excr.2000.5073 11139340

[B180] Merlo A. Casalini P. Carcangui M. L. Malventano C. Triulzi T. Menard S. (2009). FoxP3 expression and overall survival in breast cancer. J. Clin. Oncol. 27, 1746–1752 10.1200/JCO.2008.17.9036 19255331

[B181] Meyaard L. Hovenkamp E. Otto S. A. Miedema F. (1996). IL-12-induced IL-10 production by human T cells as a negative feedback for IL-12-induced immune responses. J. Immunol. 156, 2776–2782 8609396

[B182] Meyskens F. L. Jr. Kopecky K. Samson M. Hersh E. MacDonald J. Jaffe H. (1990). Recombinant human interferon gamma: adverse effects in high-risk stage I and II cutaneous malignant melanoma. J. Natl. Cancer Inst. 82, 1071–1075 10.1093/jnci/82.12.1071 2112201

[B183] Meyskens F. L. Jr. Kopecky K. J. Taylor C. W. Noyes R. D. Tuthill R. J. Hersh E. M. (1995). Randomized trial of adjuvant human interferon gamma versus observation in high-risk cutaneous melanoma: a Southwest Oncology Group study. J. Natl. Cancer Inst. 87, 1710–1713 10.1093/jnci/87.22.1710 7473820

[B184] Miller C. H. Maher S. G. Young H. A. (2009). Clinical use of interferon-gamma. Ann. N. Y. Acad. Sci. 1182, 69–79 10.1111/j.1749-6632.2009.05069.x 20074276 PMC6574079

[B185] Miyagaki T. Sugaya M. Suga H. Kamata M. Ohmatsu H. Fujita H. (2011). IL-22, but not IL-17, dominant environment in cutaneous T-cell lymphoma. Clin. Cancer Res. 17, 7529–7538 10.1158/1078-0432.CCR-11-1192 22048239

[B186] Miyao T. Floess S. Setoguchi R. Luche H. Fehling H. J. Waldmann H. (2012). Plasticity of Foxp3(+) T cells reflects promiscuous Foxp3 expression in conventional T cells but not reprogramming of regulatory T cells. Immunity 36, 262–275 10.1016/j.immuni.2011.12.012 22326580

[B187] Miyara M. Sakaguchi S. (2011). Human FoxP3^+^CD4^+^ regulatory T cells: their knowns and unknowns. Immunol. Cell Biol. 89, 346–351 10.1038/icb.2010.137 21301480

[B188] Miyara M. Yoshioka Y. Kitoh A. Shima T. Wing K. Niwa A. (2009). Functional delineation and differentiation dynamics of human CD4+ T cells expressing the FoxP3 transcription factor. Immunity 30, 899–911 10.1016/j.immuni.2009.03.019 19464196

[B189] Mizukami Y. Kono K. Kawaguchi Y. Akaike H. Kamimura K. Sugai H. (2008). CCL17 and CCL22 chemokines within tumor microenvironment are related to accumulation of Foxp3+ regulatory T cells in gastric cancer. Int. J. Cancer 122, 2286–2293 10.1002/ijc.23392 18224687

[B190] Mocellin S. Marincola F. M. Young H. A. (2005). Interleukin-10 and the immune response against cancer: a counterpoint. J. Leukoc. Biol. 78, 1043–1051 10.1189/jlb.0705358 16204623

[B191] Mocellin S. Wang E. Marincola F. M. (2001). Cytokines and immune response in the tumor microenvironment. J. Immunother. 24, 392–407 10.1097/00002371-200109000-00002 11696695

[B192] Moore K. W. de Waal Malefyt R. Coffman R. L. O’Garra A. (2001). Interleukin-10 and the interleukin-10 receptor. Annu. Rev. Immunol. 19, 683–765 10.1146/annurev.immunol.19.1.683 11244051

[B193] Moroz A. Eppolito C. Li Q. Tao J. Clegg C. H. Shrikant P. A. (2004). IL-21 enhances and sustains CD8 T cell responses to achieve durable tumor immunity: comparative evaluation of IL-2, IL-15, and IL-21. J. Immunol. 173, 900–909 15240677 10.4049/jimmunol.173.2.900

[B194] Muller A. J. Sharma M. D. Chandler P. R. Duhadaway J. B. Everhart M. E. Johnson B. A. III (2008). Chronic inflammation that facilitates tumor progression creates local immune suppression by inducing indoleamine 2, 3 dioxygenase. Proc. Natl. Acad. Sci. U.S.A. 105, 17073–17078 10.1073/pnas.0706825105 18952840 PMC2579380

[B195] Mumberg D. Monach P. A. Wanderling S. Philip M. Toledano A. Y. Schreiber R. D. (1999). CD4 T cell eliminate MHC class II-negative cancer cells in vivo by indirect effects of IFN-gamma. Proc. Natl. Acad. Sci. U.S.A. 96, 8633–8638 10.1073/pnas.96.15.8633 10411927 PMC17568

[B196] Mumm J. B. Emmerich J. Zhang X. Chan I. Wu L. Mauze S. (2011). IL-10 elicits IFNg-dependent tumor immune surveillance. Cancer Cell 20, 781–796 10.1016/j.ccr.2011.11.003 22172723

[B197] Munn D. H. Sharma M. D. Lee J. R. Jhaver K. G. Johnson T. S. Keskin D. B. (2002). Potential regulatory function of human dendritic cells expressing indoleamine 2, 3-dioxygenase. Science 297, 1867–1870 10.1126/science.1073514 12228717

[B198] Muranski P. Boni A. Antony P. A. Cassard L. Irvine K. R. Kaiser A. (2008). Tumor-specific Th17-polarized cells eradicate large established melanoma. Blood 112, 362–373 10.1182/blood-2007-11-120998 18354038 PMC2442746

[B199] Muranski P. Boni A. Wrzesinski C. Citrin D. E. Rosenberg A. Childs R. (2006). Increased intensity lymphodepletion and adoptive immunotherapy – how far can we go? Nat. Clin. Pract. Oncol. 3, 668–681 10.1038/ncpgasthep0661 17139318 PMC1773008

[B200] Muranski P. Restifo N. P. (2009). Adoptive immunotherapy of cancer using CD4 T cells. Curr. Opin. Immunol. 21, 200–208 10.1016/j.coi.2009.02.004 19285848 PMC2715842

[B201] Murphy K. M. Stockinger B. (2010). Effector T cell plasticity: flexibility in the face of changing circumstances. Nat. Immunol. 11, 674–680 10.1038/ni.1899 20644573 PMC3249647

[B202] Murugaiyan G. Saha S. (2009). Protumor vs. antitumor functions of IL-17. J. Immunol. 183, 4169–4175 10.4049/jimmunol.0901017 19767566

[B203] Muthuswamy R. Berk E. Junecko B. F. Zeh H. J. Zureikat A. H. Normolle D. (2012). NF-kB hyperactivation in tumor tissues allows tumor-selective reprogramming of the chemokine microenvironment to enhance the recruitment of cytolytic T effector cells. Cancer Res. 72, 3735–3743 10.1158/1538-7445.AM2012-SY37-02 22593190 PMC3780565

[B204] Narayanan S. Silva R. Peruzzi G. Alvarez Y. Simhadri V. R. Debell K. (2010). Human Th1 cells that express CD300a are polyfunctional and after stimulation up-regulate the T-box transcription factor eomesodermin. PLoS ONE 5:e10636 10.1371/journal.pone.0010636 20498708 PMC2869357

[B205] Nemes E. Bertoncelli L. Lugli E. Pinti M. Nasi M. Manzini L. (2010). Cytotoxic granule release dominates gag-specific CD4+ Tcell response in different phases of HIV infection. AIDS 24, 947–957 10.1097/QAD.0b013e328337b144 20179574

[B206] Nishikawa H. Kato T. Tawara I. Ikeda H. Kuribayashi K. Allen P. M. (2005). IFN-gamma controls the generation/activation of CD4+ CD25+ regulatory T cells in antitumor immune response. J. Immunol. 175, 4433–4440 16177085 10.4049/jimmunol.175.7.4433

[B207] Nishikawa H. Sakaguchi S. (2010). Regulatory T cells in tumor immunity. Int. J. Cancer 127, 759–767 20518016 10.1002/ijc.25429

[B208] Nistala K. Adams S. Cambrook H. Ursu S. Olivito B. de Jager W. (2010). Th17 plasticity in human autoimmune arthritis is driven by the inflammatory environment. Proc. Natl. Acad. Sci. U.S.A. 107, 14751–14756 10.1073/pnas.1003852107 20679229 PMC2930428

[B209] Noelle R. J. Nowak E. C. (2009). Cellular sources and immune functions of interleukin-9. Nat. Rev. Immunol. 10, 683–687 10.1038/ni0709-683 20847745 PMC3828627

[B210] Numasaki M. Fukushi J. Ono M. Narula S. K. Zavodny P. J. Kudo T. (2003). Interleukin-17 promotes angiogenesis and tumor growth. Blood 101, 2620–2627 10.1182/blood-2002-05-1461 12411307

[B211] Numasaki M. Lotze M. T. Sasaki H. (2004). Interleukin-17 augments tumor necrosis factor-induced elaboration of proangiogenic factors from fibroblasts. Immunol. Lett. 93, 39–43 10.1016/j.imlet.2004.01.014 15134897

[B212] Numasaki M. Watanabe M. Suzuki T. Takahashi H. Nakamura A. McAllister F. (2005). IL-17 enhances the net angiogenic activity and in vivo growth of human non-small cell lung cancer in SCID mice through promoting CXCR-2-dependent angiogenesis. J. Immunol. 175, 6177–6189 16237115 10.4049/jimmunol.175.9.6177

[B213] Ochi A. Nguyen A. H. Bedrosian A. S. Mushlin H. M. Zarbakhsh S. Barilla R. (2012). MyD88 inhibition amplifies dendritic cell capacity to promote pancreatic carcinogenesis via Th2 cells. J. Exp. Med. 209, 1671–1687 10.1084/jem.20111706 22908323 PMC3428946

[B214] O’Garra A. Murphy K. M. (2009). From IL-10 to IL-12: how pathogens and their products stimulate APCs to induce T(H)1 development. Nat. Immunol. 10, 929–932 10.1038/ni0909-929 19692989

[B215] O’Garra A. Vieira P. L. Vieira P. Goldfeld A. E. (2004). IL-10 producing and naturally occurring CD4 Tregs: limiting collateral damage. J. Clin. Invest. 114, 1372–1378 10.1172/JCI200423215 15545984 PMC525746

[B216] Oldenhove G. Bouladoux N. Wohlfert E. A. Hall J. A. Chou D. Dos Santos L. (2009). Decrease of Foxp3+ Treg cell number and acquisition of effector cell phenotype during lethal infection. Immunity 31, 772–786 10.1016/j.immuni.2009.10.001 19896394 PMC2814877

[B217] O’Shea J. J. Paul W. E. (2010). Mechanisms underlying lineage commitment and plasticity of helper CD4+ T cells. Science 327, 1098–1102 10.1126/science.1178334 20185720 PMC2997673

[B218] Ostrand-Rosenberg S. Sinha P. (2009). Myeloid-derived suppressor cells: linking inflammation and cancer. J. Immunol. 182, 4499–4506 10.4049/jimmunol.0802740 19342621 PMC2810498

[B219] Ouyang W. Rutz S. Crellin N. K. Valdez P. A. Hymowitz S. G. (2011). Regulation and functions of the IL-10 family of cytokines in inflammation and disease. Annu. Rev. Immunol. 29, 71–109 10.1146/annurev-immunol-031210-101312 21166540

[B220] Pagès F. Berger A. Camus M. Sanchez-Cabo F. Costes A. Molidor R. (2005). Effector memory T cells, early metastasis, and survival in colorectal cancer. N. Engl. J. Med. 353, 2654–2666 10.1056/NEJMoa051424 16371631

[B221] Palucka K. Banchereau J. (2012). Cancer immunotherapy via dendritic cells. Nat. Rev. Cancer 12, 265–276 10.1038/nrc3258 22437871 PMC3433802

[B222] Pardoll D. M. Topalian S. L. (1998). The role of CD4+ T cell responses in antitumor immunity. Curr. Opin. Immunol. 10, 588–594 10.1016/S0952-7915(98)80228-8 9794842

[B223] Pearce E. L. Mullen A. C. Martins G. A. Krawczyk C. M. Hutchins A. S. Zediak V. P. (2003). Control of effector CD8+ T cell function by the transcription factor Eomesodermin. Science 302, 1041–1043 10.1126/science.1090148 14605368

[B224] Petersson M. Charo J. Salazar-Onfray F. Noffz G. Mohaupt M. Qin Z. (1998). Constitutive IL-10 production accounts for the high NK sensitivity, low MHC class I expression, and poor transporter associated with antigen processing (TAP)-1/2 function in the prototype NK target YAC-1. J. Immunol. 161, 2099–2105 9725200

[B225] Platanias L. C. Uddin S. Bruno E. Korkmaz M. Ahmad S. Alsayed Y. (1999). CrkL and CrkII participate in the generation of the growth inhibitory effects of interferons on primary hematopoietic progenitors. Exp. Hematol. 27, 1315–1321 10.1016/S0301-472X(99)00109-5 10428508

[B226] Porter D. L. Levine B. L. Kalos M. Bagg A. June C. H. (2011). Chimeric antigen receptor-modified T cells in chronic lymphoid leukemia. N. Engl. J. Med. 365, 725–733 10.1056/NEJMoa1103849 21830940 PMC3387277

[B227] Purwar R. Schlapbach C. Xiao S. Kang H. S. Elyaman W. Jiang X. (2012). Robust tumor immunity to melanoma mediated by interleukin-9-producing T cells. Nat. Med. 18, 1248–1254 10.1038/nm.2856 PMC3518666 22772464

[B228] Putheti P. Awasthi A. Popoola J. Gao W. Strom T. B. (2010). Human CD4 memory T cells can become CD4+IL-9+ T cells. PLoS ONE 5:e8706 10.1371/journal.pone.0008706 20090929 PMC2806834

[B229] Quezada S. A. Peggs K. S. Curran M. A. Allison J. P. (2006). CTLA4 blockade and GM-CSF combination immunotherapy alters the intratumor balance of effector and regulatory T cells. J. Clin. Invest. 116, 1935–1945 10.1172/JCI27745 16778987 PMC1479425

[B230] Quezada S. A. Simpson T. R. Peggs K. S. Merghoub T. Vider J. Fan X. (2010). Tumor-reactive CD4+ T cells develop cytotoxic activity and eradicate large established melanoma after transfer into lymphopenic hosts. J. Exp. Med. 207, 637–650 10.1084/jem.20091918 20156971 PMC2839156

[B231] Qui H. Z. Hagymasi A. T. Bandyopadhyay S. St. Rose M. Ramanarasimhaiah R. Ménoret A. (2011). CD134 plus CD137 dual costimulation induces Eomesodermin in CD4 T cells to program cytotoxic Th1 differentiation. J. Immunol. 187, 3555–3564 10.4049/jimmunol.1101244 21880986 PMC3178659

[B232] Qureshi O. S. Zheng Y. Nakamura K. Attridge K. Manzotti C. Schmidt E. M. (2011). Trans-endocytosis of CD80 and CD86: a molecular basis for the cell-extrinsic function of CTLA-4. Science 332, 600–603 10.1126/science.1202947 21474713 PMC3198051

[B233] Rao R. R. Li Q. Odunsi K. Shrikant P. A. (2010). The mTOR kinase determines effector versus memory CD8+ T cell fate by regulating the expression of transcription factors T-bet and Eomesodermin. Immunity 32, 67–78 10.1016/j.immuni.2009.10.010 20060330 PMC5836496

[B234] Reinhardt R. L. Liang H. E. Locksley R. M. (2009). Cytokine-secreting follicular T cells shape the antibody repertoire. Nat. Immunol. 10, 385–393 10.1038/nrm2688 19252490 PMC2714053

[B235] Roncarolo M. Bacchetta R. Bordignon C. Narula S. Levings M. (2001). Type 1 T regulatory cells. Immunol. Rev. 182, 68–79 10.1034/j.1600-065X.2001.1820105.x 11722624

[B236] Rosenberg S. A. Yang J. C. Sherry R. M. Kammula U. S. Hughes M. S. Phan G. Q. (2011). Durable complete responses in heavily pretreated patients with metastatic melanoma using T-cell transfer immunotherapy. Clin. Cancer Res. 17, 4550–4557 10.1158/1078-0432.CCR-11-0116 21498393 PMC3131487

[B237] Rotondi M. Lazzeri E. Romagnani P. Serio M. (2003). Role for interferon-gamma inducible chemokines in endocrine autoimmunity: an expanding field. J. Endocrinol. Invest. 26, 177–180 12739748 10.1007/BF03345149

[B238] Rubtsov Y. P. Niec R. E. Josefowicz S. Li L. Darce J. Mathis D. (2010). Stability of the regulatory T cell lineage in vivo. Science 329, 1667–1671 10.1126/science.1191996 20929851 PMC4262151

[B239] Ruegg C. Yilmaz A. Bieler G. Bamat J. Chaubert P. Lejeune F. J. (1998). Evidence for the involvement of endothelial cell integrin alphaVbeta3 in the disruption of the tumor vasculature induced by TNF and IFN-gamma. Nat. Med. 4, 408–414 10.1038/nm0498-408 9546785

[B240] Sainz-Perez A. Lim A. Lemercier B. Leclerc C. (2012). The T-cell receptor repertoire of tumor-infiltrating regulatory T lymphocytes is skewed toward public sequences. Cancer Res. 72, 3557–3569 10.1158/1538-7445.AM2012-3557 22573714

[B241] Sakaguchi S. (2008). Regulatory T cells and immune tolerance. Cell 133, 775–787 10.1016/j.cell.2008.05.009 18510923

[B242] Sakaguchi S. Miyara M. Costantino C. M. Hafler D. A. (2010). FOXP3^+^ regulatory T cells in the human immune system. Nat. Rev. Immunol. 10, 490–500 10.1038/nri2785 20559327

[B243] Salama P. Phillips M. Grieu F. Morris M. Zeps N. Joseph D. (2009). Tumor infiltrating FoxP3+ T regulatory cells show strong prognostic significance in colorectal cancer. J. Clin. Oncol. 27, 186–192 10.1200/JCO.2009.24.0150 19064967

[B244] Sallusto F. Lenig D. Förster R. Lipp M. Lanzavecchia A. (1999). Two subsets of memory T lymphocytes with distinct homing potentials and effector functions. Nature 401, 708–712 10.1038/44385 10537110

[B245] Sallusto F. Lenig D. Mackay C. R. Lanzavecchia A. (1998). Flexible programs of chemokine receptor expression on human polarized T helper 1 and 2 lymphocytes. J. Exp. Med. 187, 875–883 10.1084/jem.187.6.875 9500790 PMC2212187

[B246] Sallusto F. Mackay C. R. Lanzavecchia A. (1997). Selective expression of the eotaxin receptor CCR3 by human T helper 2 cells. Science 277, 2005–2007 10.1126/science.277.5334.2005 9302298

[B247] Santin A. D. Hermonat P. L. Ravaggi A. Bellone S. Pecorelli S. Roman J. J. (2000). Interleukin-10 increases Th1 cytokine production and cytotoxic potential in human papillomavirus-specific CD8(+) cytotoxic T lymphocytes. J. Virol. 74, 4729–4737 10.1128/JVI.74.10.4729-4737.2000 10775611 PMC111995

[B248] Sariava M. O’Gara A. (2010). The regulation of IL-10 production by immune cells. Nat. Rev. Immunol. 10, 170–181 10.1038/nri2711 20154735

[B249] Schaefer G. Venkataraman C. Schindler U. (2001). Cutting edge: FISP (IL-4-induced secreted protein), a novel cytokine-like molecule secreted by Th2 cells. J. Immunol. 166, 5859–5863 11342597 10.4049/jimmunol.166.10.5859

[B250] Schreiber R. D. Old L. J. Smyth M. J. (2011). Cancer immunoediting: integrating immunity’s roles in cancer suppression and promotion. Science 331, 1565–1570 10.1126/science.1203486 21436444

[B251] Seo N. Hayakawa S. Tokura Y. (2002). Mechanisms of immune privilege for tumor cells by regulatory cytokines produced by innate and acquired immune cells. Semin. Cancer Biol. 12, 291–300 10.1016/S1044-579X(02)00015-9 12147203

[B252] Seyerl M. Kirchberger S. Majdic O. Seipelt J. Jindra C. Schrauf C. (2010). Human rhinoviruses induce IL-35-producing Treg via induction of B7-H1 (CD274) and sialoadhesin (CD169) on DC. Eur. J. Immunol. 40, 321–329 10.1002/eji.200939527 19950173

[B253] Sfanos K. S. Bruno T. C. Maris C. H. Xu L. Thoburn C. J. DeMarzo A. M. (2008). Phenotypic analysis of prostate-infiltrating lymphocytes reveals TH17 and Treg skewing. Clin. Cancer Res. 14, 3254–3261 10.1158/1078-0432.CCR-07-5164 18519750 PMC3082357

[B254] Sharma M. D. Baban B. Chandler P. Hou D. Y. Singh N. Yagita H. (2007). Plasmacytoid dendritic cells from mouse tumor-draining lymph nodes directly activate mature Tregs via indoleamine 2, 3-dioxygenase. J. Clin. Invest. 117, 2570–2582 10.1172/JCI31911 17710230 PMC1940240

[B255] Shedlock D. J. Shen H. (2003). Requirement for CD4 T cell help in generating functional CD8 T cell memory. Science 300, 337–339 10.1126/science.1082305 12690201

[B256] Shevach E. M. (2009). Mechanisms of Foxp3^+^ T regulatory cell-mediated suppression. Immunity 30, 636–645 10.1016/j.immuni.2009.04.010 19464986

[B257] Sonnenberg G. F. Fouser L. A. Artis D. (2011). Border patrol: regulation of immunity, inflammation and tissue homeostasis at barrier surfaces by IL-22. Nat. Immunol. 12, 383–390 10.1038/nrg3010 21502992

[B258] Steinbrink K. Jonuleit H. Muller G. Schuler G. Knop J. Enk A. H. (1999). Interleukin-10-treated human dendritic cells induce a melanoma antigen-specific anergy in CD8 T cells resulting in a failure to lyse tumor cells. Blood 93, 1634–1642 10029592

[B259] Steinman R. M. Banchereau J. (2007). Taking dendritic cells into medicine. Nature 449, 419–426 10.1038/nature06175 17898760

[B260] Steinman R. M. Mellman I. (2004). Immunotherapy: bewitched, bothered, and bewildered no more. Science 305, 197–200 10.1126/science.1099688 15247468

[B261] Strauss L. Bergmann C. Szczepanski M. Gooding W. Johnson J. T. Whiteside T. L. (2007). A unique subset of CD4+CD25highFoxp3+ T cells secreting interleukin-10 and transforming growth factor-beta1 mediates suppression in the tumor microenvironment. Clin. Cancer Res. 13, 4345–4354 10.1158/1078-0432.CCR-07-0472 17671115

[B262] Strutt T. M. McKinstry K. K. Kuang Y. Bradley L. M. Swain S. L. (2012). Memory CD4+ T-cell-mediated protection depends on secondary effectors that are distinct from and superior to primary effectors. Proc. Natl. Acad. Sci. U.S.A. 109, 2551–2561 10.1073/pnas.1205894109 PMC3458385 22927425

[B263] Stuller K. A. Cush S. S. Flano E. (2010). Persistent herpesvirus infection induces a CD4 T cell response containing functionally distinct effector populations. J. Immunol. 184, 3850–3856 10.4049/jimmunol.1090040 20208003 PMC3044337

[B264] Stuller K. A. Flano E. (2009). CD4 T cells mediate killing during persistent gammaherpesvirus 68 infection. J. Virol. 83, 4700–4703 10.1128/JVI.02240-08 19244319 PMC2668489

[B265] Sun J. C. Bevan M. J. (2003). Defective CD8 T cell memory following acute infection without CD4 T cell help. Science 300, 339–342 10.1126/science.1083317 12690202 PMC2778341

[B266] Sun J. C. Williams M. A. Bevan M. J. (2004). CD4+ T cells are required for the maintenance, not programming, of memory CD8+ T cells after acute infection. Nat. Immunol. 5, 927–933 10.1038/ni1105 15300249 PMC2776074

[B267] Suto A. Wurster A. L. Reiner S. L. Grusby M. J. (2006). IL-21 inhibits IFN-gamma production in developing Th1 cells through the repression of Eomesodermin expression. J. Immunol. 177, 3721–3727 16951332 10.4049/jimmunol.177.6.3721

[B268] Szabo S. J. Kim S. T. Costa G. L. Zhang X. Fathman C. G. Glimcher L. H. (2000). A novel transcription factor, T-bet, directs Th1 lineage commitment. Cell 100, 655–669 10.1016/S0092-8674(00)80702-3 10761931

[B269] Szabo S. J. Sullivan B. M. Peng S. L. Glimcher L. H. (2003). Molecular mechanisms regulating Th1 immune responses. Annu. Rev. Immunol. 21, 713–758 10.1146/annurev.immunol.21.120601.140942 12500979

[B270] Taga K. Mostowski H. Tosato G. (1993). Human interleukin-10 can directly inhibit T-cell growth. Blood 81, 2964–2971 8499633

[B271] Takahashi H. Numasaki M. Lotze M. T. Sasaki H. (2005). Interleukin-17 enhances b-FGF, HGF- and VEGF-induced growth of vascular endothelial cells. Immunol. Lett. 98, 189–193 10.1016/j.imlet.2004.11.012 15860217

[B272] Takeda K. Smyth M. J. Cretney E. Hayakawa Y. Kayagaki N. Yagita H. (2002). Critical role for tumor necrosis factor-related apoptosis-inducing ligand in immune surveillance against tumor development. J. Exp. Med. 195, 161–169 10.1084/jem.20011171 11805143 PMC2193611

[B273] Tan M. C. Goedegebuure P. S. Belt B. A. Flaherty B. Sankpal N. Gillanders W. E. (2009). Disruption of CCR5-dependent homing of regulatory T cells inhibits tumor growth in a murine model of pancreatic cancer. J. Immunol. 182, 1746–1755 19155524 10.4049/jimmunol.182.3.1746 PMC3738070

[B274] Tanikawa T. Wilke C. M. Kryczek I. Chen G. Y. Kao J. Núñez G. (2012). Interleukin-10 ablation promotes tumor development, growth, and metastasis. Cancer Res. 72, 420–429 10.1158/0008-5472.CAN-10-4627 22123924 PMC3261323

[B275] Tao M. Li B. Nayini J. (2001). In vivo effects of IL-4, IL-10 and amifostine on cytokine production in patients with acute myelogenous leukemia. Leuk. Lymphoma 41, 161–168 10.3109/10428190109057966 11342369

[B276] Tatsumi T. Kierstead L. S. Ranieri E. Gesualdo L. Schena F. P. Finke J. H. (2002). Disease-associated bias in T helper type 1 (Th1)/Th2 CD4^+^ T cell responses against MAGE-6 in HLA-DRB1*0401^+^ patients with renal cell carcinoma or melanoma. J. Exp. Med. 196, 619–628 10.1084/jem.20012142 12208877 PMC2193999

[B277] Taylor J. J. Jenkins M. K. (2011). CD4 memory T cell survival. Curr. Opin. Immunol. 23, 319–323 10.1016/j.coi.2011.03.010 21524898

[B278] Tilg H. van Montfrans C. van den Ende A. Kaser A. van Deventer S. J. Schreiber S. (2002). Treatment of Crohn’s disease with recombinant human interleukin 10 induces the proinflammatory cytokine interferon gamma. Gut 50, 191–195 10.1136/gut.50.2.191 11788558 PMC1773093

[B279] Tosolini M. Kirilovsky A. Mlecnik B. Fredriksen T. Mauger S. Bindea G. (2011). Clinical impact of different classes of infiltrating T cytotoxic and helper cells (Th1, Th2, Treg, Th17) in patients with colorectal cancer. Cancer Res. 71, 1263–1271 10.1158/0008-5472.CAN-10-2907 21303976

[B280] Trapani J. A. Smyth M. J. (2002). Functional significance of the perforin/granzyme cell death pathway. Nat. Rev. Immunol. 2, 735–747 10.1038/nri911 12360212

[B281] Van Der Bruggen P. Zhang Y. Chaux P. Stroobant V. Panichelli C. Schultz E. S. (2002). Tumor-specific shared antigenic peptides recognized by human T cells. Immunol. Rev. 188, 51–64 10.1034/j.1600-065X.2002.18806.x 12445281

[B282] van Leeuwen E. M. Remmerswaal E. B. Vossen M. T. Rowshani A. T. Wertheim-van Dillen P. M. van Lier R. A. (2004). Emergence of a CD4+CD28- granzyme B+, cytomegalovirus-specific T cell subset after recovery of primary cytomegalovirus infection. J. Immunol. 173, 1834–1841 15265915 10.4049/jimmunol.173.3.1834

[B283] Veldhoen M. Uyttenhove C. van Snick J. Helmby H. Westendorf A. Buer J. (2008). Transforming growth factor-beta “reprograms” the differentiation of T helper 2 cells and promotes an interleukin 9-producing subset. Nat. Immunol. 9, 1341–1346 10.1038/ni.1659 18931678

[B284] Vesely M. D. Kershaw M. H. Schreiber R. D. Smyth M. J. (2011). Natural innate and adaptive immunity to cancer. Annu. Rev. Immunol. 29, 235–271 10.1146/annurev-immunol-031210-101324 21219185

[B285] Vicari A. P. Trinchieri G. (2004). Interleukin-10 in viral diseases and cancer: exiting the labyrinth? Immunol. Rev. 202, 223–236 10.1111/j.0105-2896.2004.00216.x 15546396

[B286] Vignali D. A. A. (2012). Mechanisms of T_reg_ suppression: still a long way to go. Front. Immunol. 3:191 10.3389/fimmu.2012.00191 22783262 PMC3389608

[B287] Vignali D. A. A. Collison L. W. Workman C. J. (2008). How regulatory T cells work. Nat. Rev. Immunol. 8, 523–532 10.1038/nri2343 18566595 PMC2665249

[B288] Vukmanovic-Stejic M. Zhang Y. Cook J. E. Fletcher J. M. McQuaid A. Masters J. (2006). Human CD4^+^ CD25^hi^ Foxp3^+^ regulatory T cells are derived by rapid turnover of memory populations in vivo. J. Clin. Invest. 116, 2828–2836 10.1172/JCI28941 PMC1555646 16955142

[B289] Walter E. A. Greenberg P. D. Gilbert M. J. Finch R. J. Watanabe K. S. Thomas E. D. (1995). Reconstitution of cellular immunity against cytomegalovirus in recipients of allogeneic bone marrow by transfer of T-cell clones from the donor. N. Engl. J. Med. 333, 1038–1044 10.1056/NEJM199510193331603 7675046

[B290] Wang L. Yi T. Kortylewski M. Pardoll D. M. Zeng D. Yu H. (2009). IL-17 can promote tumour growth through an IL-6–Stat3 signaling pathway. J. Exp. Med. 206, 1457–1464 10.1084/jem.20081678 19564351 PMC2715087

[B291] Whiteside T. L. (2010). Immune responses to malignancies. J. Allergy Clin. Immunol. 125, S272–S283 10.1016/j.jaci.2009.09.045 20061007 PMC3721350

[B292] Wilhelm C. Turner J. Van Snick J. Stockinger B. (2012). The many lives of IL-9: a question of survival? Nat. Immunol. 13, 637–640 10.1038/ni.2303 22713829

[B293] Wilke C. M. Bishop K. Fox D. Zou W. (2011). Deciphering the role of Th17 cells in human disease. Trends Immunol. 32, 603–611 10.1016/j.it.2011.08.003 21958759 PMC3224806

[B294] Wilke C. M. Wu K. Zhao E. Wang G. Zou W. (2010). Prognostic significance of regulatory T cells in tumor. Int. J. Cancer 127, 748–758 20473951 10.1002/ijc.25464

[B295] Wilson N. J. Boniface K. Chan J. R. McKenzie B. S. Blumenschein W. M. Mattson J. D. (2007). Development, cytokine profile and function of human interleukin 17-producing helper T-cells. Nat. Immunol. 8, 950–957 10.1038/nrg2199 17676044

[B296] Wing J. B. Sakaguchi S. (2012). Multiple Treg suppressive modules and their adaptability. Front. Immunol. 3:178 10.3389/fimmu.2012.00178 22754556 PMC3386489

[B297] Wolf A. M. Wolf D. Steurer M. Gastl G. Gunsilius E. Grubek-Loebenstein B. (2003). Increase in regulatory T cells in the peripheral blood of cancer patients. Clin. Cancer Res. 9, 606–612 12576425

[B298] Wolf D. Wolf A. M. Rumpold H. Fiegl H. Zeimet A. G. Muller-Holzner E. (2005). The expression of the regulatory T cell-specific forkhead box transcription factor FoxP3 is associated with poor prognosis in ovarian cancer. Clin. Cancer Res. 11, 8326–8331 10.1158/1078-0432.CCR-05-1244 16322292

[B299] Woo E. Y. Chu C. S. Goletz T. J. Schlienger K. Yeh H. Coukos G. (2001). CD4/CD25+ T cells in tumors from patients with early-stage non-small lung cancer and late stage ovarian cancer. Cancer Res. 61, 4766–4772 11406550

[B300] Wrzesinski C. Paulos C. M. Gattinoni L. Palmer D. C. Kaiser A. Yu Z. (2007). Hematopoietic stem cells promote the expansion and function of adoptively transferred antitumor CD8 T cells. J. Clin. Invest. 117, 492–501 10.1172/JCI30414 17273561 PMC1783812

[B301] Wu K. Kryczek I. Chen L. Zou W. Welling T. H. (2009a). Kupffer cell suppression of CD8 T cells in human hepatocellular carcinoma is mediated by B7-H1/programmed death-1 interactions. Cancer Res. 69, 8067–8075 10.1158/0008-5472.CAN-08-3821 19826049 PMC4397483

[B302] Wu S. Rhee K. Albesiano E. Rabizadeh S. Wu X. Yen H. R. (2009b). A human colonic commensal promotes colon tumorigenesis via activation of T helper type 17 T cell responses. Nat. Med. 15, 1016–1022 10.1038/nm.2015 19701202 PMC3034219

[B303] Xiao M. Wang C. Zhang J. Li Z. Zhao X. Qin Z. (2009). IFN-g promotes papilloma development by up-regulating Th17-associated inflammation. Cancer Res. 69, 2010–2017 10.1158/0008-5472.SABCS-2010 19244111

[B304] Xie Y. Akpinarli A. Maris C. Hipkiss E. L. Lane M. L. Kwon E. M. (2010). Naive tumor-specific CD4+ T cells differentiated in vivo eradicate established melanoma. J. Exp. Med. 207, 651–667 10.1084/jem.20091921 20156973 PMC2839147

[B305] Xu X. Fu X. Y. Plate J. Chong A. S. (1998). IFN-γ induces cell growth inhibition by Fas-mediated apoptosis: requirement of STAT1 protein for up-regulation of Fas and FasL expression. Cancer Res. 58, 2832–2837 9661898

[B306] Yamaguchi T. Wing J. B. Sakaguchi S. (2011). Two modes of immune suppression by Foxp3(+) regulatory T cells under inflammatory or non-inflammatory conditions. Semin. Immunol. 23, 424–430 10.1016/j.smim.2011.10.002 22055883

[B307] Yang Y. Xu J. Niu Y. Bromberg J. S. Ding Y. (2008). T-bet and eomesodermin play critical roles in directing T cell differentiation to Th1 versus Th17. J. Immunol. 181, 8700–8710 19050290 10.4049/jimmunol.181.12.8700 PMC2834216

[B308] Yao W. Tepper R. S. Kaplan M. H. (2011). Predisposition to the development of IL-9-secreting T cells in atopic infants. J. Allergy Clin. Immunol. 128, 1357–1360 10.1016/j.jaci.2011.06.019 21798577 PMC3208121

[B309] Yao X. Ahmadzadeh M. Lu Y. C. Liewehr D. J. Dudley M. E. Liu F. (2012). Levels of peripheral CD4(+)FoxP3(+) regulatory T cells are negatively associated with clinical response to adoptive immunotherapy of human cancer. Blood 119, 5688–5696 10.1182/blood-2011-10-386482 22555974 PMC3382928

[B310] Ye Z. Zhou Q. Yin W. Yuan M. Yang W. Xiang F. (2012). Interleukin 22-producing CD4+ T cells in malignant pleural effusion. Cancer Lett. 326, 23–32 10.1016/j.canlet.2012.07.013 22809567

[B311] Ye Z. J. Zhou Q. Gu Y. Qin S. Ma W. Xin J. (2010). Generation and differentiation of IL-17-producing CD4+ T cells in malignant pleural effusion. J. Immunol. 185, 6348–6354 10.4049/jimmunol.1001648 20952674

[B312] Zaidi M. R. Merlino G. (2011). Two faces of IFN-g in cancer. Clin. Cancer Res. 17, 6118–6124 10.1158/1078-0432.CCR-11-0482 21705455 PMC3186825

[B313] Zamarron B. F. Chen W. J. (2011). Dual roles of immune cells and their factors in cancer development and progression. Int. J. Biol. Sci. 7, 651–658 21647333 10.7150/ijbs.7.651 PMC3107473

[B314] Zeng R. Spolski R. Finkelstein S. E. Oh S. Kovanen P. E. Hinrichs C. S. (2005). Synergy of IL-21 and IL-15 in regulating CD8+ T cell expansion and function. J. Exp. Med. 201, 139–148 10.1084/jem.20041057 15630141 PMC2212766

[B315] Zhang W. Chen Y. Wei H. Zheng C. Sun R. Zhang J. (2008). Antiapoptotic activity of autocrine interleukin-22 and therapeutic effects of interleukin-22-small interfering RNA on human lung cancer xenografts. Clin. Cancer Res. 14, 6432–6439 10.1158/1078-0432.CCR-08-0469 18927282

[B316] Zheng W. Flavell R. A. (1997). The transcription factor GATA-3 is necessary and sufficient for Th2 cytokine gene expression in CD4 T cells. Cell 89, 587–596 10.1016/S0092-8674(00)80240-8 9160750

[B317] Zhu J. Guo L. Watson C. J. Hu-Li J. Paul W. E. (2001). Stat6 is necessary and sufficient for IL-4’s role in Th2 differentiation and cell expansion. J. Immunol. 166, 7276–7281 11390477 10.4049/jimmunol.166.12.7276

[B318] Zhu J. Yamane H. Paul W. E. (2010). Differentiation of effector CD4 T cell populations. Annu. Rev. Immunol. 28, 445–489 10.1146/annurev-immunol-030409-101212 20192806 PMC3502616

[B319] Zou W. (2005). Immunosuppressive networks in the tumour environment and their therapeutic relevance. Nat. Rev. Cancer 5, 263–274 10.1038/nrc1586 15776005

[B320] Zou W. Chen L. (2008). Inhibitory B7-family molecules in the tumour microenvironment. Nat. Rev. Immunol. 8, 467–477 10.1038/nri2326 18500231

[B321] Zou W. Restifo N. P. (2010). TH17 cells in tumour immunity and immunotherapy. Nat. Rev. Immunol. 10, 248–256 10.1038/nri2742 20336152 PMC3242804

